# Research Progress on Sesquiterpenes from the Genus *Ainsliaea*

**DOI:** 10.3390/molecules29225483

**Published:** 2024-11-20

**Authors:** Hui Zhang, Ru-Ru Sun, Ya-Feng Liu, Xin Guo, Chong-Long Li, Ze-Dong Nan, Zhi-Bo Jiang

**Affiliations:** 1 School of Chemistry and Chemical Engineering, North Minzu University, Yinchuan 750021, China; zh204017596@163.com (H.Z.); srr2375764@163.com (R.-R.S.); liuyf@nmu.edu.cn (Y.-F.L.); guoxin@nun.edu.cn (X.G.); chlong@nun.edu.cn (C.-L.L.); 2 Ningxia Key Laboratory of Solar Chemical Conversion Technology, North Minzu University, Yinchuan 750021, China; 3 Key Laboratory for Chemical Engineering and Technology, State Ethnic Affairs Commission, North Minzu University, Yinchuan 750021, China

**Keywords:** *Ainsliaea*, sesquiterpenes, nuclear magnetic resonance (NMR), structure analysis

## Abstract

Sesquiterpenes constitute the principal components of the genus *Ainsliaea*, encompassing guaiane, germacrane, eudesmane, and polymer sesquiterpene lactones types. These secondary metabolites exhibit diverse pharmacological activities, including antitumor, antibacterial, anti-inflammatory, antiviral, antioxidant, hepatoprotective, and neuroprotective effects. Through a comprehensive literature search of the Web of Science, PubMed, SciFinder, and CNKI databases, it was discovered that there are as many as 145 main sesquiterpenoids in the genus *Ainsliaea*. However, the nuclear magnetic resonance (NMR) data for the sesquiterpenes in this genus have not been systematically compiled and summarized. Therefore, this review aims to highlight the chemical structures, NMR data, and pharmacological activities of sesquiterpenes in *Ainsliaea*. By meticulously analyzing published scholarly literature, our goal is to provide a solid foundation for further exploration of new sesquiterpenes and extensive utilization of this genus.

## 1. Introduction

*Ainsliaea* is a perennial herb, with over seventy species mainly distributed in southeast Asia. In China, there are forty-four species and four varieties. The majority of these plants are found around the Yangtze River Basin, with only one species distributed in the northeast. They are typically harvested in summer and autumn, and the entire plants are used for Chinese medicinal purposes [1]. According to the ‘*Supplements to the Compendium of Materia Medica*’, it is documented that Ainsliaea has a sweet and mild taste, cold properties, and belongs to the lung, spleen, and large-intestine meridians. The ‘*Comprehensive Dictionary of Chinese Herbal Medicine*’ states that *Ainsliaea* has the functions of clearing heat, promoting diuresis, cooling blood, and detoxification. *Ainsliaea fragrans* Champ. Is the primary ingredient in the national protected Chinese medicine ‘Xingxiang Tu’erfeng’ herbal granules and herbal tablets.

Since the 1980s, several studies have been conducted on the chemical composition and pharmacological activities of the genus *Ainsliaea*, leading to the discovery of over 400 compounds. The chemical constituents of this genus mainly include sesquiterpenoids, triterpenoids, steroids and their derivatives, phenolic acids, flavonoids, anthraquinones, coumarins, lignans, essential oils, and other components. Chemical studies have revealed that sesquiterpenes are the characteristic components of *Ainsliaea* plants [2,3]. The investigation has shown that sesquiterpenes in *Ainsliaea* plants mainly consist of guaiane, germacrane, eudesmane, polymer sesquiterpene lactones, and others [4]. However, NMR spectroscopy data for these sesquiterpenes derivatives have not been reported. This paper aims to provide references for the analysis and identification of new structural compounds by summarizing the ^1^H- and/or ^13^C-NMR data of 145 sesquiterpenes from the genus *Ainsliaea* between 1979 and 2022 through consulting the relevant literature.

## 2. Guaiane-Type Sesquiterpene

**Figure 1:**
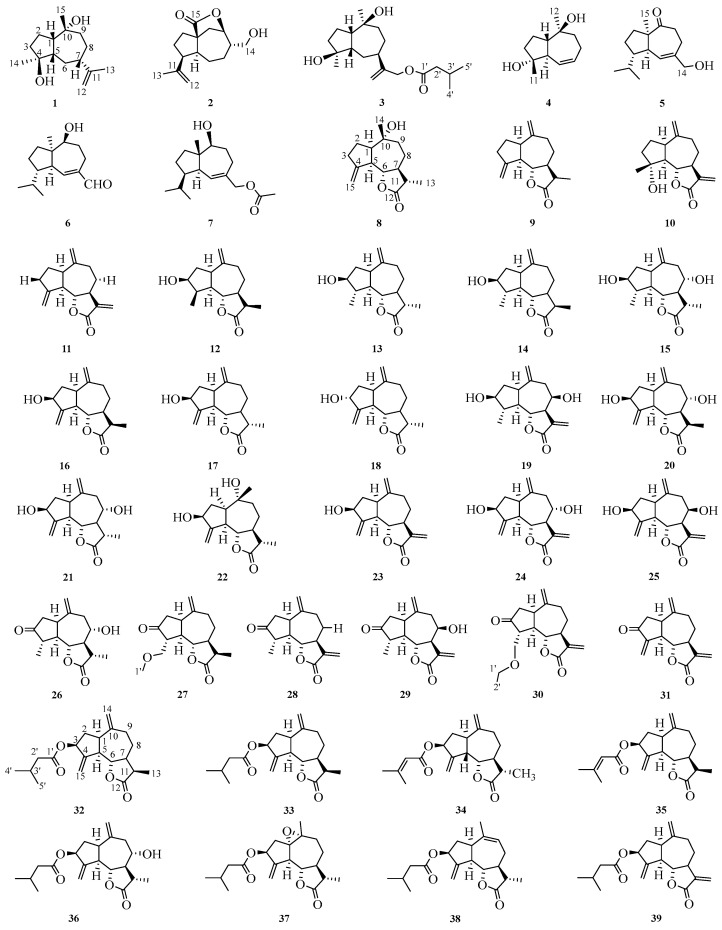

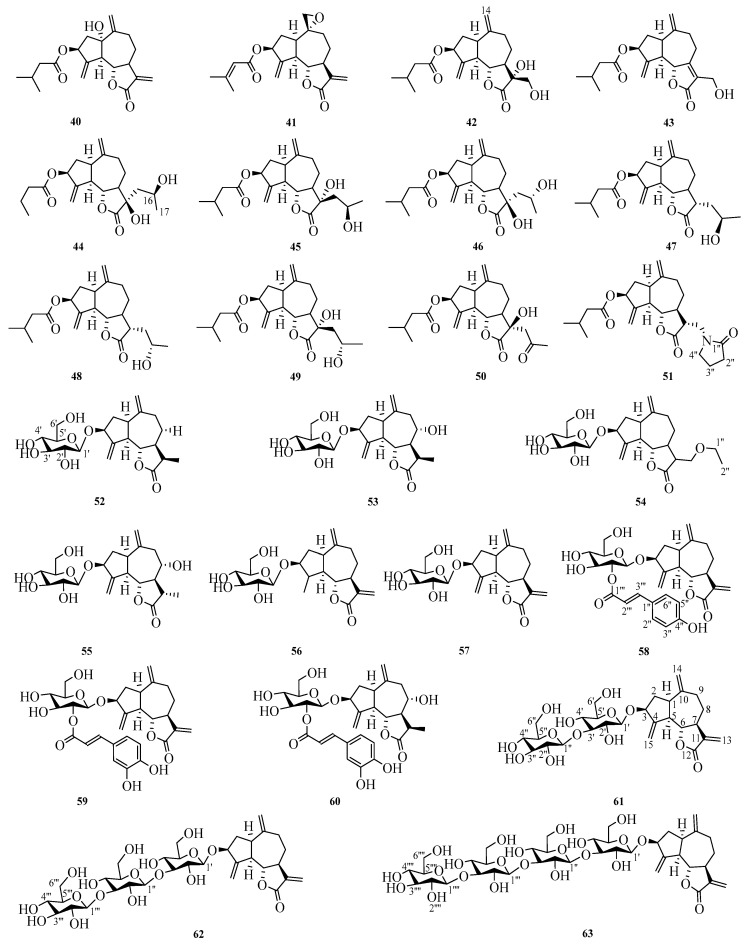
Chemical structures for compounds **1**–**63**.

Guaiane sesquiterpenes are a class of compounds with three isoprene units consisting of 5,7 fused rings, which are substituted by 4,10-dimethyl-7-isopropyl moieties as the basic nucleus. These compounds possess antibacterial, anti-inflammatory, antitumor, neuroprotective, and other biological activities [5]. Thus far, a total of 63 guaiane sesquiterpenes have been reported in this genus, mainly 12,6 guaiacan-type sesquiterpene lactones. The structures and detailed information are listed in Figure 1 and Table 1.

### 2.1. NMR Data of Guaiane Sesquiterpenes (***1***–***63***)

The ^1^H and ^13^C NMR spectroscopy results were summarized in Table 2, Table 3, Table 4, Table 5, Table 6, Table 7, Table 8, Table 9, Table 10 and Table 11. Additionally, this paper provides a summary of the nuclear magnetic data testing instrument used for compounds **1**–**63**. NMR data for compounds **2**, **8**, **12**, **27**, and **41** were obtained with Bruker AV-400 HD spectrometers (Bruker, Byersbin, Switzerland). The ^1^H and ^13^C data of compounds **3**, **7**, **36**–**38**, **42**–**49**, and **51** were obtained by a Bruker Ascend-500 spectrometer (Bruker, Nasdaq, New York, NY, USA). For compounds **11**, **25**, **55**, and **59**, the NMR data were recorded on a JEOL FX-90Q spectrometer (JEOL, Tokyo, Japan). Compounds **20**, **52**, **53**, and **57** had their ^1^H and ^13^C data taken with a Varian Mercury Plus 400 instrument (Varian, Palo Alto, CA, USA). Compounds **23**, **61**, and **62** were measured by a Varian Inova 400 instrument (Varian, Palo Alto, CA, USA). The ^1^H and ^13^C data of compounds **61**, **62**, and **63** were recorded using a unity Bruker AV500 instrument (Bruker, Switzerland). NMR data of compounds **9** and **57** were obtained using the Bruker AMX 500 (Bruker, Zurich, Switzerland) and Varian Unity Inova 500 instruments (Varian, USA). The ^1^H and ^13^C data of compounds **30** and **35** were run on a Bruker Avance 600 spectrometer (Bruker, Germany). The ^1^H- and ^13^C-NMR data of compounds **32** and **39** were collected by a Bruker DRX-500 spectrometer (Bruker, Switzerland). Nuclear magnetic data of compounds **1**, **4**, **14**, **15**, **16**, **18**, **28**, **31**, **33**, **54**, **56**, **58**, and **60** were recorded on the following instruments: VNS-600 (Varian, Palo Alto, CA, USA), Bruker ACF-500 NMR (Bruker, Germany), Bruker Avance DRX 500, Bruker Avance II 800 (Bruker, Switzerland), Bruker ARX-300 NMR (Bruker, Switzerland), Bruker Avance 400 (Bruker, Zug, Switzerland), Varian Inova 500 (Varian, Palo Alto, CA, USA), Bruker AC 200 (Bruker, Karlsruhe, Germany), Bruker Advance 500 (Bruker, Germany), Bruker AV-600 (Bruker, Switzerland), Varian 500 MHz (Varian, Palo Alto, CA, USA), and Bruker AV500-III (Bruker, Switzerland), Varian VNS600 (Varian, USA), Bruker Avance 300 (Bruker, Switzerland), and Bruker Avance 500 (Bruker, Switzerland). The ^1^H and ^13^C spectrums of compounds **5**–**6** were tested at 360 and 25 MHz, respectively; **10**, **24**, and **40** were run at 400MHz; **13** and **17** were recorded at 200 MHz for ^1^H and 50 MHz for ^13^C NMR; The ^1^H-NMR spectra of **19** and **29** were measured on 500.13 MHz; **21** and **26** were tested in the 270 MHz; **22** was taken with 300 MHz; **34** and **50** were collected in the 500 MHz. The carbon spectrum of compound **24** was determined at 100 MHz, compound **19** was recorded at 125.76 MHz, and compound **34** was recorded at 125 MHz. Carbon spectrum data for compounds **10**, **21**, **22**, **26**, **40**, and **50** have not been reported in the literature.

### 2.2. Bioactivity of Guaiane Sesquiterpenes

#### 2.2.1. Anti-Inflammatory

Nitric oxide (NO) is a related target of inflammation, and inhibiting the release of NO can treat inflammatory diseases. Dihydroestafiatol (**13**), zaluzanin C (**23**), and dehydrozaluzanin C (**31**) strongly inhibited the production of nitric oxide in RAW264.7 macrophages stimulated with lipopolysaccharide (LPS), with IC_50_ values of 7.11, 2.50, and 0.82 µM [31]. From the bioassay results, three exocyclic double bonds in guaianolides play a key role in the inhibition of the production of nitric oxide (NO), and a reduction in exocyclic double bonds will lower the inhibitory effect. Under the condition of the presence of three exocyclic double bonds, the hydroxylization of C-1 will enhance the inhibitory activity. Zaluzanin C (**23**) showed a potent inhibitory effect against NO production in LPS-stimulated RAW264.7 macrophages with an IC_50_ value of 6.54 ± 0.16 μM [34]. It may be speculated that the *α*-methylene-*γ*-lactone moiety of zaluzanin C (**23**) has a key role in its inhibition of NO release. Moreover, other functional groups, especially hydroxyl, have a great influence on the inhibitory effect of NO production. Zaluzanin C (**23**) showed remarkable inhibition against NO release in LPS-induced RAW264.7 macrophages, possibly because zaluzanin C (**23**) had an *α*-methylene-*γ*-lactone moiety and the large isovaleroxyl at C-3 hinders the binding of the compound to related proteins [37]. Guailactone can be structurally modified to obtain compounds containing the *α*-methylene-*γ*-lactone part. It is also used to introduce hydroxyl groups into guaiacols containing three outer-ring double bonds to enhance the inhibitory ability of these compounds against NO production and achieve anti-inflammatory effects.

Ainslide C (**41**), ainsliaolide A (**34**), diaspanolide B (**39**), zaluzanin C (**23**), and estafiatone (**28**) inhibit NLRP3 inflammasome activity by inhibiting the LDH release rate. Meanwhile, a Western blot assay showed that compound **41** inhibited the activity of inflammasome by inhibiting the production of Caspase-1 and IL-1*β* induced by LPS and Nigericin. Among them, the substituents of compounds **41**, **34**, **39**, **23**, and **28** are terminal double bonds, and the *α*-methylene-*γ*-butyrolactone structure seems to be the key to inhibiting LDH release activity [7]. Glucozaluzanin C (**57**) and dihydroestafiatol (**13**) showed significant anti-inflammatory activity by inhibiting the expression of nuclear factor kappa B (NF-κB) in the 293-NF-κB-luciferase reporter cell line and the production of TNF-*α*, IL-1*β*, IL-6, and IL-10 in RAW264.7 macrophages induced by lipopolysaccharide (LPS) [47].

8*α*-Hydroxy-11*α*,13-dihydrozaluzanin C (**20**) showed moderate COX-1-inhibiting activity with an IC_50_ value of 78.8 μM, comparable to that of the representative anti-inflammatory drug aspirin with an IC_50_ value of 77.2 μM. 8*α*-Hydroxy-11*α*,13-dihydrozaluzanin C (**20**) and 2′-O-E-Caffeoyl-8*α*-hydroxy-11*α*,13-dihydro-3-*β*-O-*β*-D-glucozaluzanin C (**60**) displayed potent COX-2 inhibitory activities with IC_50_ values ranging from 12.5 to 57.9 μM, in comparison with that of aspirin with an IC_50_ value of 87.6 μM [45].

#### 2.2.2. Antitumor and Cytotoxic

8-Epidesacylcinaropicrin (**25**) exhibited moderate activity toward the human tumor cell lines MDA-MB-231 and HepG2, with IC_50_ values of 18.91, and 11.16 μM, respectively [8]. Mokko lactone (**9**), zaluzanin C (**23**), and glucozaluzanin C (**57**) showed non-specific significant cytotoxicity against the A549 (non-small cell lung adenocarcinoma), SK-OV-3 (ovarian), SK-MEL-2 (skin melanoma), XF498 (CNS), and HCT15 (colon) cell lines with ED_50_ values ranging from 0.36 to 5.54 μg/mL [12]. Dehydrozaluzanin C (**31**) is a guaiacol lactone, which has significant cytotoxicity to RAW264.7 macrophages. In the presence of three outer-ring double bonds, the carbonylation of C-1 may result in a high cytotoxicity to RAW264.7 macrophages [31].

#### 2.2.3. Antiobesity

Ainsliaside A (**59**) isolated from Ainsliaea acerifolia had significant inhibitory activity on pancreatic lipase with a semi-inhibitory concentration of 15.3 ± 0.7 μM. In addition, ainsliaside A (**59**) also exhibited potent inhibitory effects against 3T3-L1 adipocyte cells and can be used as a potential antiobesity agent [43].

## 3. Germacrane-Type Sesquiterpenes

**Figure 2:**
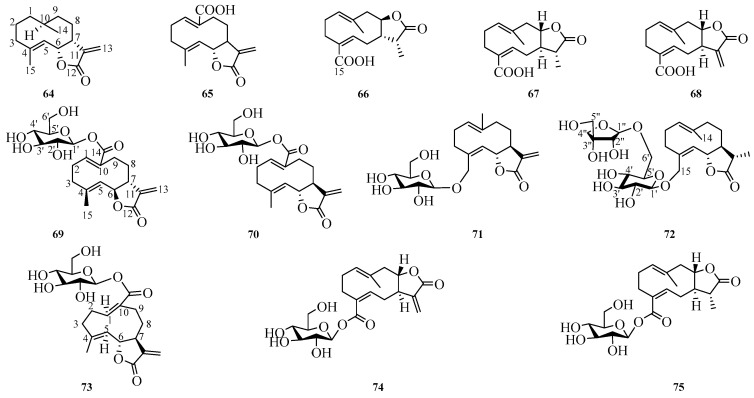
Chemical structures for compounds **64**–**75**.

Germacrane sesquiterpenes represent a class of sesquiterpenes that are extensively distributed in Compositae plants, characterized by the formation of a substantial ten-membered ring structure at the 5 and 10 positions. Currently, all twelve germacrane-type sesquiterpenes reported from this genus are lactones, with lactone rings predominantly located at the C-6, C-7 and C-7, C-8 positions. Detailed information is presented in Figure 2 and Table 12.

### 3.1. NMR Data of Germacrane-Type Sesquiterpene (***64***–***75***)

The NMR spectrum data for both ^1^H and ^13^C are presented in Table 13 and Table 14. A summary of the test instruments used to obtain the NMR data for compounds **64**–**75** is provided. The ^1^H and ^13^C data of compounds **67**, **68**, and **74** were measured with the Bruker Avance III-500 instrument (Bruker, Switzerland). The nuclear magnetic data of compounds **69** and **71** were obtained by the JEOL FX-90Q instrument (JEOL, Duzhao, Japan). The NMR spectra of compounds **65**, **66**, **67**, **72**, **73**, and **75** were recorded on various instruments including the Brukerspeckospin AC-600P (Bruker, Germany), Bruker Ascend-500 spectrometer (Bruker, Germany), Bruker Advance 500 (Bruker, Germany), Bruker Avance 600 (Bruker, Biel, Switzerland), JNM-FX-100 (JEOL, Japan), and Bruker Avance-500 (Bruker, Karlsruhe, Germany), respectively. The ^1^H-NMR data of compound **64** were measured at a frequency of 300 MHz; however, no literature reports exist regarding the proton data for compound **70**. The carbon spectra for compounds **64** and **70** were acquired at 75 MHz and 25.2 MHz, respectively.

### 3.2. Bioactivity of Germacrane-Type Sesquiterpene

Isodihydrocostunolide (**64**) showed moderate cytotoxicity against the human cancer cell lines MDA-MB-231 (IC_50_ = 18.2 μM) and HepG2 (IC_50_ = 12.2 μM), respectively [11]. Ainsliaea latifolia A (**75**), isolated from Ainsliaea latifolia, exhibited moderate activity against the HCT116 and SMMC-7721 human tumor cell lines when adriamycin was used as the positive control, with IC_50_ values of 14.72 and 10.53 μM [52].

## 4. Eudesmane Sesquiterpenes

**Figure 3:**
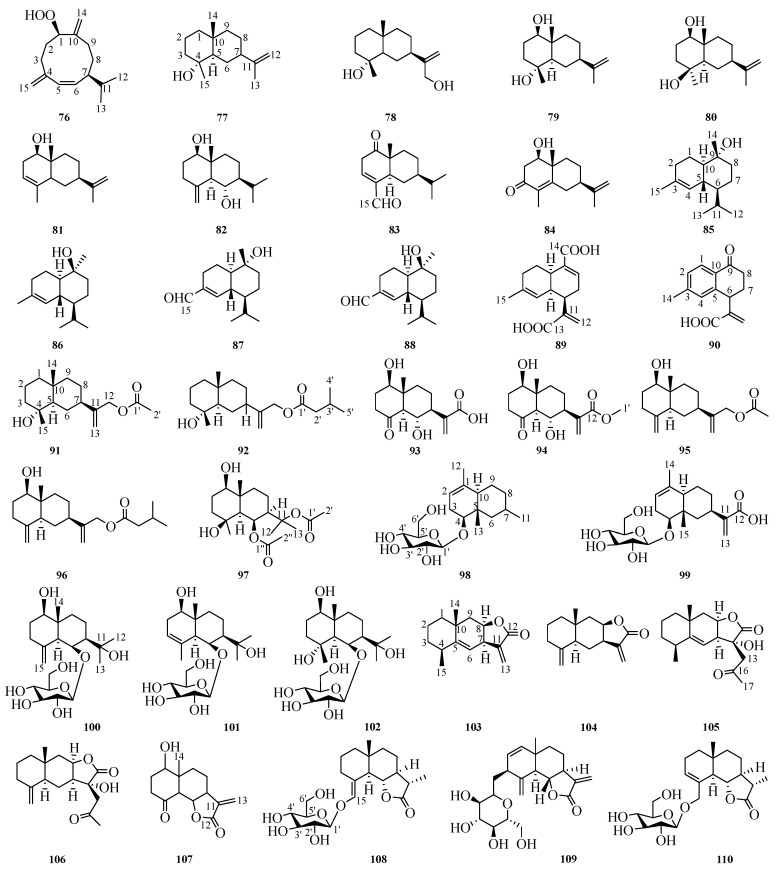
Chemical structures for compounds **76**–**110**.

The basic skeleton of eudesmane-type sesquiterpenes consists of two six-membered rings comprising a total of 15 carbon atoms. These compounds exhibit a diverse range of biological activities, including anti-inflammatory, cytotoxic, antibacterial, antimalarial, insecticidal, and neuroprotective activities [53]. Up to now, 35 eudesmane-type sesquiterpenes have been reported within this genus. Their structures and detailed information are shown in Figure 3 and Table 15.

### 4.1. NMR Data of Eudesmane-Type Sesquiterpene (***76***–***110***)

The ^1^H and ^13^C spectrum data are shown in Table 16, Table 17, Table 18, Table 19 and Table 20. An overview of the testing instruments used for the NMR data of compounds **76**–**110** is provided. The NMR data for compounds **82** and **87** were measured with the Bruker DRX-500 spectrometer (Bruker, Germany). The ^1^H and ^13^C spectra of compounds **85** and **86** were obtained by the JEOL MN 100 instrument (JEOL, Japan). For compounds **87** and **88**, NMR data were recorded on the Bruker AM 400 spectrometer (Bruker, Switzerland). The NMR data for compounds **93**, **98**, and **99** were taken with the Varian 500 (Varian, Palo Alto, CA, USA) and Bruker AV500-III instruments (Bruker, Switzerland). Compounds **97**, **108**, and **110** had their NMR data obtained on the Bruker AV-600 spectrometer (Bruker, Switzerland). The ^1^H and ^13^C data of compounds **100**, **101**, **102**, and **109** were measured using the JEOL FX-90Q instrument (JEOL, Japan). The NMR data for compounds **100**, **101**, and **102** were recorded by the GSX-270 (JEOL, Japan) and GSX-500 NMR instruments (JEOL, Japan). The ^1^H and ^13^C spectra of compounds **103** and **104** were analyzed using the Bruker Avance DRX 500 spectrometer (Bruker, Germany). NMR data for compounds **76**, **77**, **79**, **80**, **83**, **85**, **92**, **94**, **95**, and **96** were obtained from various instruments including Bruker AMX 500 (Bruker, Zurich, Switzerland) and Varian Unity Inova 500 (Varian, Palo Alto, CA, USA), Bruker DRX-300 (Bruker, Karlsruhe, Germany), JEOL JNM LA-500 (JEOL, Japan), Varian Mercury-300 BB (Varian, San Jose, USA), Varian Mercury Plus 400 (Varian, USA), NT-200 (University of California, Davis, CA, USA), Bruker Advance 500 (Bruker, Germany), Bruker Avance 600 (Bruker, Biel, Switzerland), Bruker AV-400 HD (Bruker, Byersbin, Switzerland), Bruker Ascend 500 (Bruker, Zurich, Switzerland), and other instruments. Compound **78** was measured at 200 MHz for ^1^H NMR. Compounds **84** and **107** were tested at 400 MHz. Compounds **89** and **90** were recorded at 600 MHz. Compound **91** was run at 60 MHz. Compounds **105** and **106** were operated at 500 MHz. No test instrumentation has been reported in the literature regarding NMR spectrum data for compound **81**. Furthermore, no hydrogen spectrum data of compound **86** have been reported in the existing literature. The carbon spectrum data for compound **84** were measured at 50.32 MHz; the ^13^C data collection for compound **107** occurred at 100 MHz; compound **86** was recorded at 75 MHz for ^13^C NMR; while the carbon spectrum data of compounds **89** and **90** were measured at 150 MHz. Compounds **78**, **91**, **105**, and **106** have not been described within any available literature concerning their ^13^C NMR spectra.

### 4.2. Bioactivity of Eudesman-Type Sesquiterpene

Eucalyptane sesquiterpenes exert anti-inflammatory effects by inhibiting the activity of the NLRP3 inflammasome and inhibiting the production of NO in RAW264.7 macrophages. Cyperusol C (**79**) derived from Ainsliaea pertyoides can inhibit NLRP3 inflammasome activity by inhibiting the LDH release rate [7]. Ainsliatone B (**94**) strongly inhibited the production of nitric oxide in RAW264.7 macrophages stimulated with lipopolysaccharide (LPS), with IC_50_ values of 8.78 µM [31].

Isoalantolactone (**104**) remarkably inhibited the proliferation of MGC803 cell lines with IC_50_ values of 2.2 ± 0.2 μM. Double-bond moieties may be necessary for its cytotoxicity [34]. Alantolactone (**103**) exhibited significant inhibition against the human tumor cell lines A549, HCT116, MGC803, and CCRF-CEM with IC_50_ values of 3.56, 2.23, 2.89, and 14.67 µM, respectively [39].

## 5. Polymer Sesquiterpene Lactones

**Figure 4:**
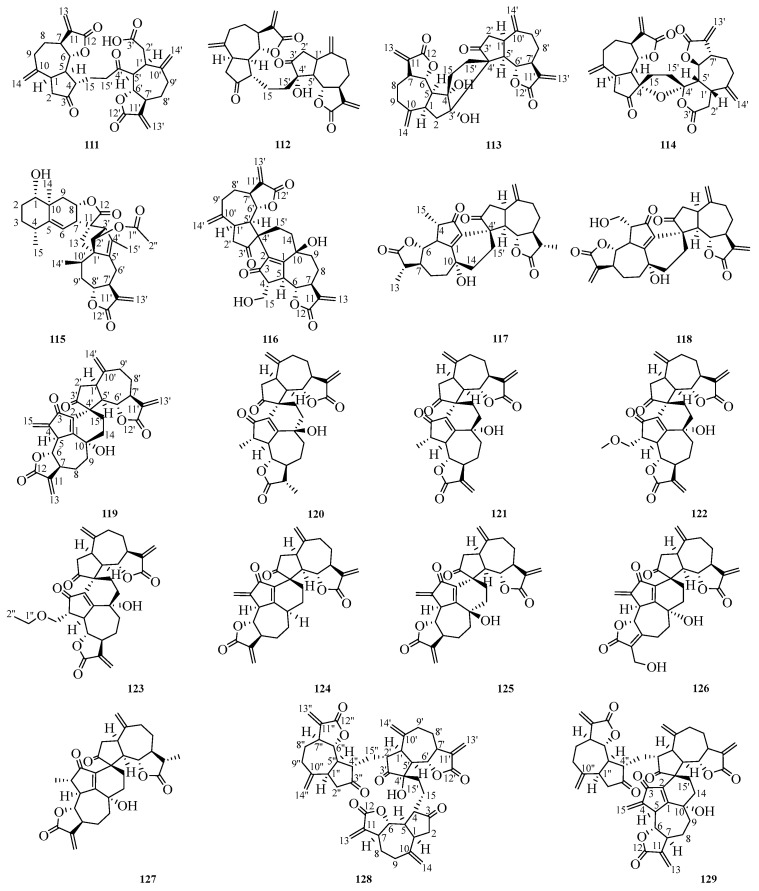

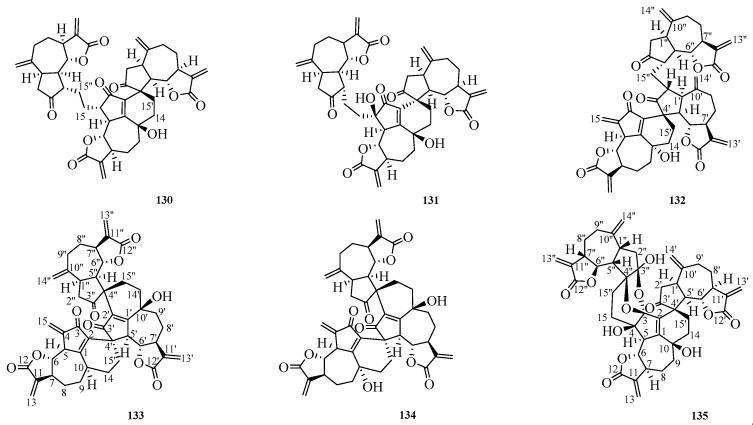
Chemical structures for compounds **111**–**135**.

In Ainsliaea plants, polymer sesquiterpene lactones are typically formed through the polymerization of two or three sesquiterpene units; most monomeric precursors belong to guaiane sesquiterpenes. The structures of the 25 reported polymer sesquiterpene lactones can be found in Figure 4 and Table 21.

### 5.1. NMR Data of Polymer Sesquiterpene Lactones (***111***–***135***)

The ^1^H and ^13^C NMR spectroscopy are summarized in Table 22, Table 23, Table 24, Table 25 and Table 26. This paper also provides an overview of the nuclear magnetic resonance testing instruments used for compounds **111**–**135**. The ^1^H and ^13^C spectra of compounds **111**, **112**, **114**, **130**, and **131** were measured using the Bruker Avance III-500 instrument. The NMR data for compounds **113**, **117**, **118**, **120**, **121**, **122**, and **123** were recorded on the Bruker Avance 600 spectrometer. For compounds **115** and **116**, the respective ^1^H and ^13^C spectra were obtained by the Bruker Avance 400 instrument. Compound **119**’s NMR data were acquired on the Bruker AC 200 spectrometer. NMR data for compounds **119**, **124**, and **125** were acquired using the Bruker AV500 instrument. The ^1^H and ^13^C data of compounds **126**, **127**, and **132** were taken with the Bruker Avance AV500 spectrometer. Data for compounds **128**, **129**, and **135** were performed on the Bruker Avance III-600 instrument. NMR data collection for compounds **133** and **134** was conducted using the Bruker Avance 400 instrument.

### 5.2. Bioactivity of Polymer Sesquiterpene Lactones

#### 5.2.1. Antitumor and Cytotoxic

Polymer sesquiterpene lactones inhibit the activity of tumor cell lines or cancer cell lines. The dimeric sesquiterpene lactone japonicone A (**115**) showed significant inhibitory activity against the three tested human tumor cell lines 95D, MDA-MB-231, and HepG2, with IC_50_ values of 9.10, 3.82, and 1.43 μM, respectively [8]. Ainsliatriolide C (**131**), ainsliadimer B (**116**), and ainsliatrimer B (**134**) were isolated from Ainsliaea yunnanensis and showed very significant selective cytotoxic activities on MDA-MB-468, PANC-1, HEPG2, and A549 cells, and IC_50_ values from 5.1 μM to 34.4 μM [81]. With DOX (doxorubicin) as the positive control, the antitumor activity of the isolated compounds against A549, LOVO, CEM, and MDA-MB-M-435 (MDA) was detected by an MTT assay. Both Ainsliatrimer A (**133**) and ainsliatrimer B (**134**) showed potent cytotoxicites against the LOVO and CEM cell lines [75]. Ainsliatriolide A (**130**) and ainsliatriolide B (**135**) exhibited stronger cytotoxicity on A-549, HT-29, BEL-7402, and HL-60 cancer cell lines, especially ainsliatriolide A (**130**) which displayed potent cytotoxicity with an averaged IC_50_ value of 1.17 μM against four cancer cell lines [80].

With cisplatin as a positive control, the cytotoxicity of the compounds isolated from Ainsliaea fragrans was tested in the five cancer cell lines of C6 rat glioma cells, Huh1, HCC-LM3 human hepatocellular carcinoma cells, PANC-1 human pancreatic cells, and Hela human cervical cancer cells. The cytotoxicity results showed that ainsfragolide (**132**) is an unusual guaianolide sesquiterpene trimer, which is generated by a new C2″–C15″ bond and has a significant inhibitory effect on five cancer cells with a half-inhibitory concentration value in the range of 0.4–8.3 μM. Three trimers ainsfragolide (**132**), ainsliatrimer A (**133**), and ainsliatrimer B (**134**) showed more potent cytotoxic effect against the five test cancer cells than the dimers. When compared with ainsliatrimer B (**134**), the decreased activity of ainsliatrimer A (**133**) indicated that the introduction of an extra hydroxy group at the C-10 position is important for the resultant cytotoxicity. Similarly, the dimers gochnatiolide A (**119**) and gochnatiolide B (**125**) with a C-10 OH group were more cytotoxic than gochnatiolide C (**124**), respectively. Furthermore, gochnatiolide A (**119**) with a β-configuration of OH-10 was about 2–10-fold more active than gochnatiolide B (**125**) with α-OH at C-10. In contrast, ainsliadimer B (**116**) with 10β–OH and OH-15 groups showed reduced activity [78]. Therefore, we can try to obtain a trimer with a β-configuration hydroxyl at the C-10 position and no hydroxyl at the C-15 position by synthesis or structural modification, so as to improve the cytotoxic activity of polymer sesquiterpene lactones against cancer cells.

#### 5.2.2. Anti-Inflammatory

Macrocephadiolide A (**114**) and macrocephadiolide B (**111**) showed a potent inhibitory effect on nitric oxide (NO) production, with IC_50_ values of 0.99 and 6.13 µM, respectively, on lipopolysaccharide (LPS)-stimulated RAW264.7 macrophages. Macrocephadiolide A (**114**) dose-dependently suppressed the expression of inducible NO oxidase (iNOS) through inhibiting nuclear factor kappa B (NF-κB) activation [72]. Ainsliadimer A (**113**) represents an unusual carbon skeleton with a cyclopentane system connecting the two monomeric sesquiterpene lactone units. This unique molecule exerted potent inhibitory activity against the production of nitric oxide in RAW264.7 cells stimulated by LPS, with an IC_50_ value of 2.41 µg/mL [73]. Gochnatiolide A (**119**) showed significant anti-inflammatory activity by inhibiting the expression of nuclear factor kappa B (NF-κB) in the 293-NF-κB-luciferase reporter cell line and the production of TNF-α, IL-1β, IL-6, and IL-10 in RAW264.7 macrophages induced by lipopolysaccharide (LPS) [47]. The anti-inflammatory effects of polymer sesquiterpene lactones in Ainsliaea are achieved by inhibiting the production of nitric oxide (NO) in RAW264.7 macrophages, inhibiting the activity of nuclear factor kappa B (NF-κB) in the luciferase 293-NF-κB-luciferase reporter cell line, and inhibiting the expression of TNF-α, IL-1β, IL-6, and IL-10 in RAW264.7 macrophages.

#### 5.2.3. Other Biological Activities

The polymer sesquiterpene lactones also have antibacterial and blood-sugar regulation effects. Ainsliatrimer B (**134**) also showed a medium inhibiting effect on Bacillus subtilis with a MIC value of 32 μg/mL [81]. Macrocephatriolide B (**128**) showed potent inhibition against protein tyrosine phosphatase 1B (PTP1B) with an IC_50_ value of 26.26 ± 0.88 μM. In insulin-stimulated C2C12 myotubes, macrocephatriolide B (**128**) dose-dependently enhanced glucose uptake by activating the insulin signaling pathway and might represent a new scaffold of insulin sensitizers [79].

## 6. Other Sesquiterpenoids

**Figure 5:**
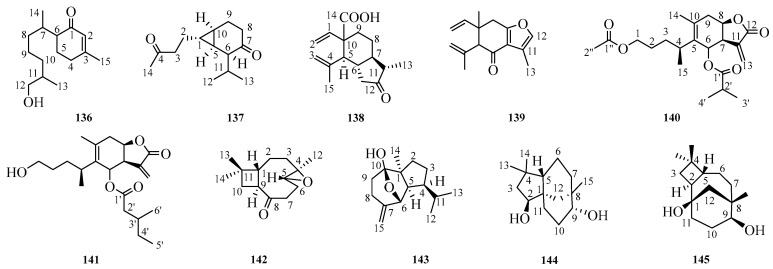
Chemical structures for compounds **136**–**145**.

Beyond those mentioned above, there exist other categories of sesquiterpenoids and their derivatives, such as myrrhanes, lananes, and carabanes. And further details can be referenced in Figure 5 and Table 27.

### 6.1. NMR Data of Other Sesquiterpenoids ***136***–***145***

The NMR spectra of ^1^H and ^13^C are summarized in Table 28 and Table 29. This paper also compiles data regarding the nuclear magnetic resonance testing instruments used for the compounds numbered from **136** to **145**. The ^1^H and ^13^C data of compound **136** were recorded using a Bruker instrument operating at 300 MHz. For compound **137**, NMR data collection was performed with the Bruker-Avance-III-400 instrument (Bruker, Switzerland). For compound **140**, the NMR data were conducted on a Bruker AMX 400 instrument (Bruker, Zurich, Switzerland). The NMR data for compound **141** were taken with a Bruker Avance-500 instrument (Bruker, Switzerland). Both the ^1^H and ^13^C data of compound **143** were measured using JEOL Lamda 400 (JEOL, Japan) and Lamda 600 instruments (JEOL, Japan). Finally, compounds **144** and **145** had their respective ^1^H and ^13^C data collected via the JEOL JNM-GX400 instrument (JEOL, Japan). The ^1^H data for compounds **138**, **139**, and **142** were measured at frequencies of 600 MHz, 300 MHz, and 400 MHz, respectively. The ^13^C NMR of compounds **138**, **139**, and **142** were tested under 150 MHZ, 75 MHZ, and 100 MHZ, respectively.

### 6.2. Bioactivity of Other Sesquiterpenoids

At the concentration of 10 μMol/L, ainsliaea acid A (**138**) significantly inhibited nuclear factor kappa B (NF-κB) in lipopolysaccharides-induced 293-NF-κB-luciflucidase reporter cell lines with a inhibitory rate of 17.5%. Further experiments showed that ainsliaea acid A (**138**) exerted anti-inflammatory effects by inhibiting the production of tumor necrosis factor-α (TNF-α), interleukin-1β (IL-1β), IL-6, and IL-10 in RAW264.7 macrophages induced by LPS [67].

## 7. Conclusions

Sesquiterpenoids derived from plants of the genus Ainsliaea exhibit a wealth of pharmacological activities, demonstrating significant antitumor, anti-inflammatory, antibacterial, and antiobesity effects. Currently, the existing literature primarily focuses on the chemical composition and pharmacological effects of these plants; however, comprehensive NMR data summarizing the related components remain scarce. This paper consolidates the ^1^H- and/or ^13^C-NMR data for sesquiterpenes extracted from *Ainsliaea* species, and the pharmacological activities of sesquiterpenes are summarized, thereby providing a valuable reference for discovering novel sesquiterpenes and differentiating between various types. It also offers essential data support for structural analysis and compound identification. Additionally, exploring new sesquiterpene constituents is crucial for investigating their pharmacodynamic material basis, as well as their structure–activity relationships and mechanisms of action, and enriching the diversity of natural products.

The sesquiterpenoids found in the plants of Ainsliaea are mostly guaiane and eudesmane, and the active ingredients found are mostly guaiane and polymer sesquiterpene lactones. Guaiacanolactone containing a *α*-methylene-*γ*-lactone moiety and guaiac lactones containing hydroxyl groups with three outer-ring double bonds have a significant inhibitory effect on NO production. Trimeric sesquiterpene lactones with a *β*-configuration hydroxyl at the C-10 position and without a hydroxyl group at C-15 have high cytotoxic activity. Therefore, it is of great significance to find compounds containing these components in natural products or to obtain these compounds through synthesis and structural modification to improve the anti-inflammatory activity of guaiane sesquiterpenes and the cytotoxic activity of polymer sesquiterpene lactones. The limited amount of germacrane and other sesquiterpenes reported within this genus renders the data support for structural analysis and identification of these compounds insufficiently convincing. It is essential to conduct further investigations into their chemical constituents, identify additional sesquiterpenes, and enhance the diversity of sesquiterpene types within the genus. Moreover, a comprehensive summary of the NMR spectral data pertaining to triterpenoids, steroids and their derivatives, phenolic acids, flavonoids, anthraquinones, coumarins, lignans, and other components remains lacking and needs to be further summarized.

## Figures and Tables

**Table 1:** The compound name, molecular formula, and NMR test reagent of guaiane-type sesquiterpene.

No.	Compound Name	Molecular Formula	Solvent	Ref.
**1**	Epi-guaidiol A	C_15_H_26_O_2_	CD_3_OD	[6]
**2**	Ainslide A	C_15_H_22_O_3_	CDCl_3_	[7]
**3**	Spicatene B	C_20_H_34_O_4_	CDCl_3_	[8]
**4**	4*β*,10*α*-Dimethyl-1*β*,5*α*-bicycle[3,5,0]dec-6-en-4*α*,10*β*-diol	C_12_H_20_O_2_	CDCl_3_	[9]
**5**	Aphanamol I	C_15_H_24_O_2_	CDCl_3_	[10]
**6**	Aphanamol II	C_15_H_24_O_2_	CDCl_3_	[10]
**7**	Yunnanol A	C_17_H_28_O_3_	CDCl_3_	[11]
**8**	Ainslide E	C_15_H_22_O_3_	CDCl_3_	[7]
**9**	4(15),10(14)-Guaiadien-12, 6-olide mokkolactone	C_15_H_20_O_2_	CDCl_3_	[12]
**10**	4-Hydroxy-10(14),11(13)-guaiadien-6,12-olide	C_15_H_20_O_3_	CDCl_3_	[13]
**11**	Dehydrocostuslactone	C_15_H_18_O_2_	CDCl_3_	[14]
**12**	Ainslide F	C_15_H_22_O_3_	CDCl_3_	[7]
**13**	Dihydroestafiatol	C_15_H_22_O_3_	CDCl_3_	[15]
**14**	4*β*,15,11*β*,13-Tetrahydrozaluzanin C	C_16_H_22_O_2_	CDCl_3_	[16]
**15**	Isolipidiol	C_15_H_22_O_4_	CD_3_OD	[17,18]
**16**	11*α*,13-Dihydrozaluzanin C	C_15_H_20_O_3_	CDCl_3_	[19]
**17**	11*β*,13-Dihydrozaluzanin C	C_15_H_20_O_3_	CDCl_3_	[15]
**18**	11*β*,13-Dihydro-3-epizaluzanin C	C_15_H_20_O_3_	CDCl_3_	[20]
**19**	8*β*-Hydroxy-4*β*, 15-dihydrozaluzanin C	C_15_H_20_O_4_	CDCl_3_	[21]
**20**	8*α*-Hydroxy-11*α*, 13-dihydrozaluzanin C	C_15_H_20_O_4_	CDCl_3_	[22]
**21**	11*β*,13-Dihydrodesacylynaropicrin	C_15_H_20_O_4_	CDCl_3_	[18,23]
**22**	l0*α*-Hydroxy-10(14),11*β*(13)-tetrahydroxaluzanin C	C_15_H_22_O_4_	CDCl_3_	[24]
**23**	Zaluzanin C	C_15_H_18_O_3_	CDCl_3_	[25]
**24**	Desacylcynaropicrin	C_15_H_18_O_4_	CDCl_3_ CD_3_OD/CDCl_3_	[18,26]
**25**	8-Epidesacylcinaropicrin	C_15_H_18_O_4_	C_5_D_5_N	[27]
**26**	Isoamberboin	C_15_H_20_O_4_	CDCl_3_	[28]
**27**	Ainslide D	C_16_H_22_O_4_	CDCl_3_	[7]
**28**	Estafiatone	C_15_H_18_O_3_	CDCl_3_	[29]
**29**	8-Epigrosheimin	C_15_H_18_O_4_	CDCl_3_	[30]
**30**	Ainsliaolide B	C_17_H_22_O_4_	CDCl_3_	[31]
**31**	Dehydrozaluzanin C	C_15_H_16_O_3_	CDCl_3_	[32]
**32**	Diaspanolide A	C_20_H_28_O_4_	CDCl_3_	[33]
**33**	Diaspanolide E	C_20_H_28_O_4_	CDCl_3_	[34]
**34**	Ainsliaolide A	C_24_H_26_O_4_	CDCl_3_	[35]
**35**	Ainsliaolide D	C_20_H_26_O_4_	CDCl_3_	[36]
**36**	8*α*-Hydroxy-diaspanolide A	C_20_H_28_O_5_	CDCl_3_	[8]
**37**	Yunnanolides H	C_20_H_28_O_5_	CDCl_3_	[37]
**38**	Yunnanolides I	C_20_H_28_O_4_	CDCl_3_	[37]
**39**	Diaspanolide B	C_20_H_26_O_4_	CDCl_3_	[33]
**40**	l*α*-Hydroxy-3-O-isobutyrate	C_20_H_26_O_5_	CDCl_3_	[38]
**41**	Ainslide C	C_20_H_24_O_5_	CDCl_3_	[7]
**42**	Yunnanolide J	C_20_H_28_O_6_	CDCl_3_	[11]
**43**	Spicatene A	C_20_H_26_O_5_	CDCl_3_	[8]
**44**	Yunnanolides A	C_22_H_32_O_6_	CDCl_3_	[37]
**45**	Yunnanolides C	C_22_H_32_O_6_	CDCl_3_	[37]
**46**	Yunnanolides D	C_22_H_32_O_6_	CDCl_3_	[37]
**47**	Yunnanolides E	C_22_H_32_O_5_	CDCl_3_	[37]
**48**	Yunnanolides F	C_22_H_32_O_5_	CDCl_3_	[37]
**49**	Yunnanolides B	C_22_H_32_O_6_	CDCl_3_	[37]
**50**	Pertyolide C	C_22_H_30_O_6_	CDCl_3_	[39]
**51**	Yunnanolides G	C_24_H_33_NO_5_	CDCl_3_	[37]
**52**	11*α*, 13-Dihydroglucozaluzanin C	C_21_H_30_O_8_	CD_3_OD/C_5_D_5_N	[22]
**53**	8*α*-Hydroxy-11*α*, 13-dihydroglucozaluzanin C	C_21_H_30_O_9_	CD_3_OD	[22]
**54**	13-Ethoxy-4(15),10(14)-dien-guai-6,12-olide-3-O-*β*-D-glucopyranoside	C_23_H_35_O_9_	CD_3_OD	[40]
**55**	11*β*,13-Dihydro-8*α*-hydroxyglucozaluzanin C	C_21_H_30_O_9_	C_5_D_5_N	[41]
**56**	4*β*,15-Dihydrozaluzanin C	C_21_H_30_O_8_	DMSO	[42]
**57**	Glucozaluzanin C	C_21_H_28_O_8_	CDCl_3/_C_5_D_5_N/ CD_3_SOCD_3_	[12,22]
**58**	Ainsliaside C	C_30_H_34_O_10_	CD_3_OD	[43]
**59**	Ainsliaside A	C_30_H_34_O_11_	C_5_D_5_N	[44]
**60**	2′-O-E-Caffeoyl-8*α*-hydroxy-11*α*,13-dihydro-3-*β*-O-*β*-D-glucozaluzanin C	C_30_H_36_O_12_	CD_3_OD	[45]
**61**	Macrocliniside B	C_27_H_38_O_13_	DMSO CD_3_OD	[25,46]
**62**	Macrocliniside I	C_33_H_48_O_18_	DMSO	[25,46]
**63**	ZaluzaninC-3-O-*β*-glucopyranosyl-(1→3)-*β*-glucopyranosyl-(1→3)-*β*-glucopyranosyl-(1→3)-*β*-glucopyranoside	C_39_H_58_O_23_	DMSO	[46]

**Table 2:** ^1^H-NMR data of compounds **1**–**8**.

NO.	1 [6]	2 [7]	3 [8]	4 [9]	5 [10]	6 [10]	7 [11]	8 [7]
	CD_3_OD	CDCl_3_	CDCl_3_	CDCl_3_	CDCl_3_	CDCl_3_	CDCl_3_	CDCl_3_
1	2.54, ddd, 10.8, 8.4, 8.4	—	2.74, q, 9.3	2.05, m	—	—	—	2.30, q, 7.6
2*α*	1.81–1.85, m	1.94–2.02, m	1.95, m	1.76, dd, 11.7, 6.4	2.08	—	1.63, m	1.78–1.87, m
2*β*	1.51–1.54, m	1.59–1.70, m	1.49, m	1.66, dd, 11.7, 7.3	1.35	—	1.42, m	1.90–1.98, m
3*α*	1.67, br d, 10.4	2.47–2.60, m	1.69, m	1.70, dd, 7.3, 6.4	1.8	—	1.43, m	2.38–2.48, m
3*β*	1.66, dd, 10.4, 3.6	1.86–1.97, m			1.35			
4	—	2.99–3.08, m	—	—	1.66	—	1.63, m	—
5	2.00, ddd, 13.2, 10.8, 3.6	2.07, td, 12.1, 2.4	2.03, m	2.24, dd, 11.5, 2.5	2.27	—	2.03, m	2.76, t, 8.9
6*α*	1.60, dd, 13.2, 3.6	1.76–1.85, m	1.78, m	5.74, br d, 11.0	5.51	6.62, d, 5	5.44, d, 4.5	4.08, t, 9.9
6*β*	1.35, ddd, 13.2, 13.2, 10.8	1.25–1.37, m	1.64, m					
7*α*	—	1.86–1.97, m	2.14, m	5.80, ddd, 11.0, 5.3, 2.4	—	—	—	2.03–2.09, m
7*β*	2.12, ddd, 10.8, 10.8, 4.2	1.15–1.27, m	—	—	—	—	—	—
8*α*	1.72, ddd, 13.8, 7.2, 4.2, 3.6	—	1.72, m	1.98, m	2.54	—	2.17, m	1.99–2.07, m
8*β*	1.49, dddd, 13.8, 13.8 10.8, 4.2	2.13–2.22, m	1.86, m	2.30, m	2.29	—		1.30–1.39, m
9*α*	1.89, ddd, 13.8, 7.2, 4.2	4.77–4.81, m	1.66, m	1.61, ddd, 14.0, 9.5, 1.8	2.81	—	1.76, m	1.87–1.94, m
9*β*	1.61, ddd, 13.8, 13.8, 3.6	—	1.78, m	1.83, ddd, 14.0, 9.5, 2.0	2.41	—		1.58–1.67, m
10*α*	—	2.47–2.57, m	—	—	—	3.40, dd, 11, 6	3.44, dd, 10.5, 6.0	—
10*β*	—	1.95, d, 13.1	—	—	—	—	—	—
11	—	—	—	1.19, s	1.57	—	1.54, m	2.17–2.25, m
12*α*	4.65, br s	4.79–4.84, m	5.03, br s	1.26, s	0.9	0.92, d, 7	0.89, d, 7.0	—
12*β*	4.58, br s		5.01, br s					—
13	1.70, s	1.68, s	4.56, s	—	0.92	0.93, d, 7	0.89, d, 7.0	1.23, d, 6.9
14*α*	1.13, s	3.62, dd, 10.7, 4.5	1.30, s	—	4.01	9.37, s	4.49, m	1.13, s
14*β*		3.38, dd, 10.7, 7.6		—				
15*α*	1.20, s	—	1.25, s	—	1.27	1.04, s	0.97, s	5.11, s
15*β*		—		—				4.95, s
2′	—	—	2.22, m	—	—	—	2.07, s	—
3′	—	—	2.11, m	—	—	—	—	—
4′	—	—	0.97, d, 6.6	—	—	—	—	—
5′	—	—	0.97, d, 6.6	—	—	—	—	—

**Table 3:** ^1^H-NMR data of compounds **9**–**17**.

NO.	9 [12]	10 [13]	11 [14]	12 [7]	13 [15]	14 [16]	15 [17]	16 [19]	17 [15]
	CDCl_3_	CDCl_3_	CDCl_3_	CDCl_3_	CDCl_3_	CDCl_3_	C_5_D_5_N	CDCl_3_	CDCl_3_
1	2.89, dt, 8.0, 4.5	3.02, br ddd, 12.5, 8, 8	—	2.69–2.76, m	—	2.76, m	2.79, m	2.83, m	—
2*α*	1.95, m	1.82, m	—	1.92–2.06, m	—	1.75, m	2.02, m	2.34, m	—
2*β*	1.87, m		—		—	2.15, m	2.24, m		—
3*α*	2.49, m	1.82, m	—	4.21–4.28, m	3.71, m	3.72, q, 6.4	3.91, m	4.54, t, 6.0	4.54, t, 7.4
3*β*		1.92, m	—	—	—	—	—	—	—
4	—	—	—	2.31–2.39, m	—	1.85, m	2.14, m	—	—
5	2.81, br dd, 9.5, 8.0	2.38, dd	—	2.22–2.31, m	—	1.75, m	1.98, m	—	—
6	3.93, t, 9.5	4.06, dd	3.98, t, 9	4.23, t, 9.7	3.93, t, 10	3.93, t, 9.7	3.93, m	4.13, t, 9.0	4.02, t, 9.5
7	2.12, m	2.77, ddddd	—	2.26–2.33, m	—	1.85, m	2.18, ddd, 10.4, 10.0, 9.6	—	—
8*α*	1.94, m	2.26, dddd	—	1.83–1.91, m	—	2.10, m	—	1.41, m	—
8*β*	1.32, m	1.38, dddd	—	1.33–1.42, m	—	1.23, m	3.83, m		—
9*α*	2.22, dd, 12.0, 7.0	2.69, ddd	—	2.62–2.68, m	—	2.60, dt, 13.0, 4.0	2.35, dd, 12.8, 11.8	—	—
9*β*	2.05, dt, 12.0, 5.0	1.94, br ddd	—	1.78–1.88, m	—	1.85, m	3.00, dd, 12.8, 4.8	—	—
11	2.49, m	—	—	2.66–2.71, m	2.12, m	2.20, m	2.77, m	—	2.14, qd, 11, 6.9
13*α*	1.25, d, 7.0	6.24, d	6.25, d, 3.5	1.18, d, 7.8	1.21, d, 6.8	1.29, d, 7.0	1.68, d, 7.2	1.16, d, 6.0	1.23, d, 6.9
13*β*		5.53, d	5.51, d, 3.2						
14*α*	4.89, br s	5.01, br s	4.91, s	5.00, s	4.95, s	4.92, s	4.99, br s	4.95, s	4.96, s
14*β*	4.79, br s	4.97 br s	4.84, s	4.95, s			5.09, br s	4.93, s	4.93, s
15*α*	5.21, d, 2.0	1.32, s	5.29, br s	0.97, d, 7.2	1.24, d, 6.3	1.20, d, 7.3	1.44, d, 6.4	5.40, s	5.38, t, 1.9
15*β*	5.06, d, 2.0		5.09, br s					5.31, s	5.29, t, 1.9

**Table 4:** ^1^H-NMR data of compounds **18**–**25**.

NO.	18 [20]	19 [21]	20 [22]	21 [23]	22 [24]	23 [25]	24 [26]	25 [27]
	CDCl_3_	CDCl_3_	CDCl_3_	CDCl_3_	CDCl_3_	CDCl_3_	CD_3_OD	C_5_D_5_N
1	—	2.83, br ddd, 11.1, 10.6, 6.6	2.91, m	2.92, m	1.9–2.2, m	—	2.98, ddd, 10.6, 9.0, 7.1	—
2*α*	—	2.21, ddd, 13.0, 6.6, 6.6	1.71, m	2.25, m	1.9–2.2, m	—	1.73, ddd, 12.6, 11.0, 9.0	—
2*β*	—	1.79, ddd, 13.0, 10.6, 8.8	2.29, m	1.77, m	2.32, ddd, 14, 8, 8	—	2.11, ddd, 12.6, 7.2, 7.0	—
3	4.53, t, 7.6	3.75, ddd, 8.8, 8.8, 6.6	4.52, m	4.55, br d, 8	4.53, br t, 8, 1.9	4.53, m	4.50, br dd, 9.1, 7.5	—
4	—	1.90, m	—	—	—	—	—	—
5	—	1.93, m	2.88, m	2.85, m	2.72, br t, 9, 1.9	—	2.88, m	—
6	4.12, dd, 10.0, 9.2	4.27, dd, 9.7, 9.7	4.12, dd, 9.9, 9.9	4.07, t, 10	4.25, t, 9.9	4.06, t, 9.2	4.17, dd, 10.4, 8.9	—
7	—	2.80, m	2.35, m	2.00, q, 10	1.9–2.2, m	—	2.89, m	—
8*α*	—	4.34, br m	—	—	1.4–1.8, m	—	—	—
8*β*	—	—	3.78, m	3.78, t, 9, 4.5		—	3.90, ddd, 9.5, 5.0, 4.8	—
9*α*	—	2.37, dd, 13.5, 3.4	2.10, m	2.72, dd, 14, 5	1.4–1.8, m	—	2.70, dd, 13.6, 5.1	—
9*β*	—	2.69, dd, 13.5, 4.9	2.72, dd, 12.5, 4.5	2.21, dd, 14, 7		—	2.27, dd, 13.6, 4.6	—
11	—	—	2.87, m	2.58, m	2.25, dq, 12, 6.8	—	—	—
13*α*	1.14, d, 8.0	6.37, d, 3.6	1.29, d, 7.7	1.42, d, 3p, 7	1.22, d, 6.8	6.16, d, 3.2	6.15, dd, 3.2, 1.3	6.49, d, 3.5
13*β*		5.61, d, 3.2				5.46, d, 3.2	6.12, dd, 3.5, 1.3	5.68, overlapped
14*α*	4.94, s	5.16, br s	5.08, br s	5.11, br	1.17, s	4.95, br s	5.08, d, 1.7	5.70, s
14*β*	4.91, s	5.03, br s	5.01, br s	5.00, br		4.90, br s	4.97, d, 2.0	
15*α*	5.38, s	1.22, d, 6.3	5.42, dd, 1.7, 1.6	5.41, t, 1	5.24, t, 1.9	5.42, br s	5.37, d, 2.3	5.68, overlapped
15*β*	5.30, s		5.32, dd, 1.7, 1.6	5.32, t, 1	5.20, t, 1.9	5.29, br s	5.30, d, 2.1	5.25, br s

**Table 5:** ^1^H-NMR data of compounds **26**–**33**.

NO.	26 [28]	27 [7]	28 [29]	29 [30]	30 [31]	31 [32]	32 [33]	33 [34]
	CDCl_3_	CDCl_3_	CD_3_OD	CDCl_3_	CDCl_3_	CDCl_3_	CDCl_3_	CDCl_3_
1	3.12, m	3.08, td, 8.5, 3.1	3.10, td, 8.5, 2.0	3.05, ddd, 8.4, 8.0, 3.1	3.10, t, 9.0	3.12, ddd	2.89, m	2.89–2.94, m
2*α*	2.25, c	2.50–2.55, m	2.16, t, 8.5	2.60, dd, 19.3, 8.4	2.48, d, 16.8	2.68, dd	2.46, m	1.98–2.03, m
2*β*	2.25, c	2.41–2.50, m		2.54, ddd, 19.3, 3.1, 1.4	2.55, dd, 16.8, 9.0	2.56, dd		1.74–1.80, m
3	—	—	—	—	—	—	5.54, m	5.56, dd, 6.3, 2.1
4	—	2.31–2.38, m	2.30, d, 7.5	2.33, ddq, 10.3, 6.9, 1.4	2.30, m	—	—	—
5	2.5, c	2.78–2.85, m	2.31, m	2.28, ddd, 10.3, 9.2, 8.0	2.91, q, 9.0	3.27, tdd	2.83, m	2.81–2.86, m
6	3.93, t, 9	4.06, t, 9.6	4.40, 8.8	4.55, dd, 9.2, 9.2	3.98, t, 9.0	4.01, t	4.08, t, 9.8	4.10, t, 9.7
7	2.05, q, 10	2.53–2.61, m	3.00, dddd, 8.0, 8.0, 3.0, 3.0	3.16, dddd, 9.2, 3.5, 3.0, 2.0	3.01, m	3.03, m	2.67, m	2.37–2.42, m
8*α*	—	1.93–2.02, m	2.35, m	4.46, br m	1.47, m	3.03, m	—	1.87–1.94, m
8*β*	—	1.38–1.49, m	1.48, m		2.33, m	1.46, m	—	1.37–1.46, m
9*α*	2.82, dd, 13, 6	2.55–2.63, m	2.63, m	2.69, dd, 13.9, 3.0	2.22, m	2.20, m	2.46, m	2.44–2.49, m
9*β*	2.25, c	2.03–2.12, m	2.22, m	2.50, dd, 13.9, 4.2	2.61, m	2.60, m		
11*α*	—	2.71–2.80, m	—	—	—	—	—	2.66–2.72, m
11*β*	2.5, c	—	—	—	—	—	—	—
13*α*	1.44, d, 7	1.19, d, 7.7	6.30, d, 3.5	6.45, d, 3.5	5.57, d, 3.6	6.30, d	1.15, d, 7.8	1.17, d, 7.8
13*β*			5.58, d, 3.5	5.68, d, 3.0	6.29, d, 3.6	5.58, d		
14*α*	5.06, br	4.93, s	5.02, br s	5.09, br s	4.66, brs	4.94, s	4.89, d, 9.0	4.91, s
14*β*	4.76, br	4.63, s	4.69, br s	4.84, br s	4.98, brs	4.60, s		4.92, s
15*α*	1.24, d, 7	3.84, dd, 8.9, 3.1	1.28, d, 6.5	1.28, d, 6.9	3.71, dd, 9.0, 3.0	6.25, dd	5.25, t, 2.0	5.27, t, 2.1
15*β*		3.61, dd, 8.9, 3.1			3.98, dd, 9.0, 3.0	5.87, dd	5.39, t, 2.0	5.42, t, 2.2
1′	—	3.30, s	—	—	3.48, q, 7.2	—	—	—
2′	—	—	—	—	1.14, t, 7.2	—	—	2.24, dd, 7.1, 1.7
3′	—	—	—	—	—	—	—	2.09–2.17, m
4′	—	—	—	—	—	—	0.96, d, 6.6	0.98, d, 6.6
5′	—	—	—	—	—	—	0.96, d, 6.6	0.98, d, 6.6

**Table 6:** ^1^H-NMR data of compounds **34**–**42**.

NO.	34 [35]	35 [36]	36 [8]	37 [37]	38 [37]	39 [32]	40 [38]	41 [7]	42 [11]
	CDCl_3_	CDCl_3_	CDCl_3_	CDCl_3_	CDCl_3_	CDCl_3_	CDCl_3_	CDCl_3_	CDCl_3_
1	2.89, m	2.93, m	2.95, q, 8.4	—	2.18, m	2.94, m	—	2.44–2.52, m	2.90, q, 8.0
2*α*	2.46, m	2.48, m	2.44, m	2.23, m	2.67, m	1.78, m	2.43, br dd, 14, 8	2.25–2.37, m	2.49, m
2*β*	1.79, m	1.79, m	1.77, m		1.51, m		2.18, dd, 14, 7	1.52–1.62, m	1.80, m
3	5.55, dd, 8, 6	5.57, ddt, 8, 6, 2	5.56, m	5.72, m	5.54, m	5.56, m	5.75, tt, 8, 7, 1.5	5.50, t, 6.7	5.56, m
5	1.93, m	2.85, m	2.82, m	2.66, d, 11.5	2.55, m	2.85, m	2.80, br d, 10, 1.5	2.89–2.97, m	2.04, m
6	3.99, t, 9	4.11, t, 10	4.00, t, 9.9	3.88, t, 10.5	4.07, t, 10.0	4.06, dd, 16.9, 7.6	3.91, t, 10	4.22, t, 9.5	4.39, t, 10.0
7	2.81, m	2.42, m	2.01, m	1.69, m	1.76, m	2.85, m	3.05, ddddd, 10, 10, 4, 3.5, 3	2.92–3.01, m	2.76, t, 9.5
8*α*	2.11, m	1.92, m	—	1.45, m	2.40, m	—	2.30, m, 4, 4	2.24–2.33, m	1.80, m
8*β*	1.31, m	1.42, m	3.77, m		1.97, m	—	1.45, m, 10, 9	1.44–1.55, m	
9*α*	2.49, m	2.52, m	2.71, dd, 13.1, 5.0	1.45, m	5.54, m	2.46, m	2.63, ddd, 12, 9, 4	1.88–1.98, m	2.48, m
9*β*	2.03, m	2.01, m	2.23, m		—		2.30, m	1.78, dt, 14.3, 5.0	2.04, m
11	2.20, m	2.69, p, 8	2.57, m	2.32, m	2.29, m	—	—	—	—
13*α*	1.22, d, 7	1.16, d, 8	1.42, d, 7.0	1.25, m	1.23, d, 7.0	5.49, d, 3.1	6.21, d, 3.5	6.27, d, 3.0	3.79, d, 12.0
13*β*						6.21, d, 3.5	5.49, d, 3	5.55, d, 3.0	3.65, d, 11.0
14*α*	4.91, s	4.91, br s	5.05, s	1.35, s	1.80, br s	4.97, d, 6.4	5.20, br s	2.75, d, 4.4	4.93, d, 6.5
14*β*		4.92, br s	4.99, s				5.09, br s	2.54, d, 4.4	
15*α*	5.26, brt, 2	5.28, br t, 2	5.43, t, 2.2	5.50, s	5.44, br s	5.27, t, 2.1	5.53, t, 1.5	5.45, s	5.41, br t, 2.0
15*β*	5.38, brt, 2	5.41, br t, 2	5.28, t, 2.2	5.41, s	5.33, br s	5.45, t, 2.0	5.36, t, 1.5	5.25, s	5.30, br t, 2.0
2′*α*	5.71, s	5.72, br s	2.23, m	2.17, m	2.55, m	—	2.59, qq, 7, 7	5.69, s	2.23, dd, 7.5, 2.0
2′*β*					2.18, m	—			
3′	—	—	2.13, m	2.09, m	2.09, m	—	—	—	2.13, m
4′	1.89, s	1.91, br s	0.97, d, 6.6	0.95, d, 6.5	0.95, d, 6.5	0.96, d, 6.6	1.20, d	1.91, s	0.97, d, 6.5
5′	2.17, s	2.19, br s	0.97, d, 6.6	0.95, d, 6.5	0.95, d, 6.5	0.96, d, 6.6	1.19, d	2.18, s	0.97, d, 6.5

**Table 7:** ^1^H-NMR data of compounds **43**–**51**.

NO.	43 [8]	44 [37]	45 [37]	46 [37]	47 [37]	48 [37]	49 [37]	50 [39]	51 [37]
	CDCl_3_	CDCl_3_	CDCl_3_	CDCl_3_	CDCl_3_	CDCl_3_	CDCl_3_	CDCl_3_	CDCl_3_
1	2.90, m	2.86, q, 8.5	2.89, m	2.90, q, 8.5	2.90, q, 8.0	2.90, q, 3.0	2.89, m	2.88, q, 8.4	2.13, m
2*α*	2.21, m	2.44, m	2.57, dt, 13.0, 4.0	2.46, m	2.03, m	2.02, m	2.57, dt, 13.0, 4.0	2.44, m	2.44, m
2*β*	1.84, m	1.76, m	1.96, m	1.78, m	1.80, m	1.82, m	1.99, m	1.76, m	
3	5.61, t, 7.6	5.54, m	5.54, m	5.55, m	5.57, m	5.57, m	5.56, m	5.56, m	5.56, m
5	2.62, m	2.71, t, 9.5	2.89, m	2.73, t, 9.5	2.80, t, 9.5	2.82, t, 9.2	2.89, m	2.75, t, 9.2	2.79, t, 9.5
6	4.81, d, 11.2	4.29, t, 9.5	4.01, t, 9.5	4.36, t, 9.5	4.08, t, 10.0	4.05, t, 9.5	3.91, t, 9.5	4.37, t, 9.2	4.02, t, 9.5
7	—	1.87, m	2.35, m	2.07, m	2.03, m	2.13, m	2.34, m	1.94, m	2.45, m
8*α*	3.05, m	2.10, m	2.10, m	2.12, m	2.15, m	2.13, m	2.12, m	1.64, m	2.38, q, 8.0
8*β*	2.50, m		1.39, m	1.30, m	1.39, m	1.35, m	1.35, m	1.81, m	
9*α*	2.52, m	2.44, m	2.49, dt, 14.0, 8.0	2.46, m	2.47, m	2.49, m	2.50, dt, 14.0, 8.0	2.03, m	2.03, m
9*β*	2.17, m	2.01, m	1.75, m	2.07, m			1.75, m	2.50, m	1.78, dt, 14.0, 6.5
11	—	—	—	—	2.46, m	2.49, m	—	—	2.89, q, 8.0
13*α*	4.38, dd, 16.6, 13.3	—	1.76, m	1.96, m	1.80, m	1.82, m	1.76, m	2.62, d, 16.7	3.69, dd, 4.5, 4.5
13*β*		1.64, dd, 15.0, 2.5	1.68, m	1.78, m	1.69, m	1.67, m	1.57, dd, 15.0, 2.5	2.80, d, 16.7	3.57, dd, 5.0, 5.0
14*α*	5.01, s	4.91, s	4.93, s	4.93, s	4.93, s	4.92, s	4.93, s	4.93, s	4.92, s
14*β*	4.94, s	4.88, s	4.89, s	4.91, s			4.90, s	4.91, s	4.90, s
15*α*	5.42, br s	5.41, br t, 1.5	5.39, br t, 2.0	5.42, br t, 2.0	5.39, br t, 2.0	5.40, t, 2.0	5.41, br t, 2.0	5.43, br s	5.37, br t, 2.0
15*β*		5.27, br t, 1.5	5.29, br t, 2.0	5.29, br t, 2.0	5.27, br t, 2.0	5.26, t, 2.0	5.29, br t, 2.0	5.30, br s	5.25, br t, 2.0
16	—	4.97, m	4.27, m	4.16, m	3.97, m	4.21, m	4.31, m	—	—
17	—	1.22, d, 6	1.23, d, 6.0	1.30, d, 8.5	1.24, d, 6.0	1.24, d, 6.0	1.23, d, 6.0	2.32, s	—
2′	2.23, m	2.21, dd, 7.5, 1.5	2.23, dd, 7.5, 2.0	2.22, dd, 7.5, 2.0	2.22, dd, 8.0, 2.0	2.23, dd, 8.0, 1.5	2.22, dd, 8.0, 2.0	2.23, dd, 7.1, 1.7	2.23, dd, 8.0, 1.5
3′	2.11, m	2.15, m	2.10, m	1.96, m	2.15, m	2.13, m	2.12, m	2.12, m	2.13, m
4′	0.97, d, 6.6	0.95, d, 6.5	0.97, d, 6.5	0.97, d, 6.5	0.97, d, 6.5	0.97, d, 6.5	0.97, d, 6.5	0.97, d, 6.5	0.96, d, 7.0
5′	0.97, d, 6.6	0.95, d, 6.5	0.97, d, 6.5	0.97, d, 6.5	0.97, d, 6.5	0.97, d, 6.5	0.97, d, 6.5	0.97, d, 6.5	0.96, d, 7.0

Note: The ^1^H-NMR data of 2″*α*, 2″*β*, 3″, and 4″ for **51** were recorded as 2.30, m; 1.32, m; 2.03, m; and 3.45, m, respectively.

**Table 8:** ^1^H-NMR data of compounds **52**–**57**.

NO.	52 [22]	52 [22]	53 [22]	54 [40]	55 [41]	56 [42]	57 [12]	57 [22]
	CD_3_OD	C_5_D_5_N	CD_3_OD	CD_3_OD	C_5_D_5_N	DMSO	CDCl_3_	C_5_D_5_N
1	2.96, m	2.75, m	3.00, m	2.97, q, 8.4	—	2.76, m	2.80, br t, 10.0	2.77, m
2*α*	2.35, m	2.22, m	2.30, m	2.36, m, overlapped	—	1.69, dd, 12, 10	2.39, m	2.30, m
2*β*	1.96, m	1.81, m	1.96, m	1.96, dt, 14.0, 6.9	—	2.14, dd, 13.5, 7.0	1.98, m	1.92, m
3	4.62, m	4.41, m	4.61, m	4.63, dd, 7.8, 5.9	4.84, br t, 7	3.56, dd, 15.5, 8.0	4.65, br dd, 6.0, 6.0	4.81, dd, 7.3, 1.5
4	—	—	—	—	—	1.85, m	—	—
5	2.79, dd, 9.5, 9.5	2.68, m	2.83, dd, 9.9, 9.9	2.75, dd, 9.9, 8.4	—	1.91, m	3.01, dd, 17.5, 8.5	2.74, m
6	4.33, dd, 9.9, 9.5	4.33, dd, 9.9, 9.5	4.38, dd, 10.6, 9.9	4.23, t, 9.9	—	3.93, t, 10	4.28, dd, 9.0, 9.0	4.26, m
7	2.43, m	2.33, m	2.36, m	2.39, m, overlapped	—	2.78, m	2.89, m	2.70, m
8*α*	1.88, m	1.58, m		2.16, tt, 10.0, 5.0	—	2.25, m	2.28, m	1.97, m
8*β*	1.44, m	1.20, m	3.72, m	1.39, m	—	1.24, m	1.46, m	1.16, m
9*α*	2.03, m	2.10, m	2.15, dd, 12.6, 8.0	2.08, ddd, 13.7, 9.3, 5.1	—	2.54, m	2.21, m	2.10, m
9*β*	2.53, m	2.36, m	2.71, dd, 12.6, 5.0	2.52, m, overlapped	—	1.97, m	2.52, ddd, 13.0, 6.5, 6.5	2.37, m
11	2.67, m	2.64, m	2.77, m	2.54, dt, 11.8, 3.7	—	—	—	—
13*α*	1.12, dd, 7.7, 1.5	1.05, d, 7.7	1.23, d, 7.7	3.71, dd, 9.9, 3.7	1.65, d, 7	5.59, d, 2	6.12, d, 3.0	5.53, br d, 1.5
13*β*				3.63, dd, 9.9, 3.7		6.00, d, 2.5	5.57, d, 3.0	5.87, br d, 1.5
14*α*	4.99, s	5.00, s	5.09, s	4.99, s	5.14, br s	4.95, d, 5	5.01, br s	5.02, d, 1.1
14*β*	4.91, s	4.83, s	4.98, s	4.91, s	5.01, br s	4.99, d, 5	4.94, br s	4.83, d, 1.1
15*α*	5.40, s	5.82, br s	5.36, d, 1.3	5.42, d, 1.7	5.87, br s	1.15, d, 10	5.44, br s	6.23, d, 3.4
15*β*	5.31, s	5.50, br s	5.32, d, 1.3	5.30, d, 1.7	5.54, br s		5.35, br d, 1.0	5.38, d, 3.4
1′	4.46, d, 7.7	5.04, d, 7.9	4.44, d, 7.7	4.45, d, 7.8	5.06, d, 7	4.19, d, 7.5	4.47, d, 7.5	5.05, d, 7.9
2′	3.24, m	3.94, m	3.20, m	3.23, m	—	2.95, m	3.20–3.40, m	3.96, m
3′	3.36, m	4.24, m	3.31, m	3.36, m	—	3.16, m	3.87, dd, 10.0, 10.0	4.24, m
4′	3.28, m	4.06, m	3.26, m	3.28, m	—	3.04, m	3.20–3.40, m	4.08, m
5′	3.28, m	4.22, m	3.24, m	3.27, m	—	3.08, m	3.67, dd, 12.0, 5.5	4.22, m
6′*α*	3.66, dd, 11.9, 5.0	4.36, m	3.64, dd, 12.0, 6.0	3.88, dd, 12.0, 1.9	—	3.42, dd, 11.5, 6.0	3.20–3.40, m	4.40, dd, 11.8, 5.5
6′*β*	3.86, br d, 11.9	4.56, dd, 11.9, 2.0	3.87, dd, 12.0, 2.0	3.67, dd, 12.0, 5.2	—	3.65, dd, 9.5, 4.5		4.57, dd, 11.8, 2.4

Note: The ^1^H-NMR data of 1″ and 2″ for **54** were recorded as 3.51, q, 7.0 and 1.17, t, 7.0.

**Table 9:** ^1^H-NMR data of compounds **58**–**61**.

NO.	58 [43]	59 [44]	60 [45]	61 [46]	NO.	58 [43]	59 [44]	60 [45]	61 [46]
	CD_3_OD	C_5_D_5_N	CD_3_OD	DMSO		CD_3_OD	C_5_D_5_N	CD_3_OD	DMSO
1	2.99, dd, 16.8, 8.4	—	2.99, t, 9.1	—	15α	5.43, br s	5.45, br s	5.32, d, 1.1	5.38, br s
2*α*	2.33, m	—	2.26, m	—	15β	5.35, br s		5.27, d, 1.1	5.20, br s
2*β*	1.96, m	—	1.98, m	—	1′	4.45, d, 7.2	—	4.66, d, 8.1	4.40, d, 7.8
3	4.63, m	—	4.64, m	4.50, m	2′	3.87, m	5.65, br t, 10	4.85, m	—
5	2.78, dd, 9.6, 9.6	—	2.85, br d, 9.4	—	3′	3.37, t, 8.4	—	3.58, t, 8.6	—
6	4.26, dd, 9.6, 9.3	—	4.04, t, 10.2	4.13, dd, 10.2, 8.9	4′	3.28, m	—	3.38, m	—
7	2.89, m	—	2.34, dd, 8.1, 2.1	—	5′	3.26, m	—	—	—
8*α*	2.26, m	—	3.50, m	—	6′α	3.86, dd, 12.0, 2.4	—	3.91, dd, 12.0, 2.1	—
8*β*	1.45, m	—		—	6′β	3.68, dd, 12.0, 5.4	—	3.70, dd, 12.0, 5.8	—
9*α*	2.18, m	—	2.06, m	—	1″	—	—	—	4.32, d, 7.8
9*β*	2.50, m	—	2.58, dd, 11.5, 4.7	—	2″	7.56, d, 7.8	6.9–7.5, m	7.02, d, 2.0	—
11	—	—	2.72, t, 7.8	—	3″	7.37, d, 7.8	—	—	—
13*α*	6.09, d, 3.0	—	1.09, d, 7.7	6.02, d, 3.5	5″	7.37, d, 7.8	6.9–7.5, m	6.78, d, 8.1	—
13*β*	5.56, d, 3.0	—		5.61, d, 3.2	6″	7.56, d, 7.8	6.9–7.5, m	6.94, dd, 8.2, 1.9	—
14*α*	5.01, s	5.13, br s	5.11, br s	4.91, br s	2‴	6.48, d, 15.6	6.37, d, 15	6.24, d, 15.9	—
14*β*	4.91, s		4.90, br s	4.88, br s	3‴	7.58, d, 15.6	7.82, d, 15	7.55, d, 15.9	—

**Table 10:** ^1^H-NMR data of compounds **61**–**63**.

NO.	61 [25]	62 [46]	62 [25]	63 [46]	NO.	61 [25]	62 [46]	62 [25]	63 [46]
	CD3OD	DMSO	DMSO	DMSO		CD3OD	DMSO	DMSO	DMSO
1	—	—	4.88, d, 7.6	2.92, m	1′	4.54, d, 8.0	4.40, d, 7.8	5.01, d, 8.0	4.40, d, 7.8
2*α*	—	—	—	2.25, m	2′	—	—	—	3.43, m
2*β*	—	—	—	1.77, m	3′	—	—	—	3.40, m
3	4. 64, m	4.50, t, 7.4	4.64, m	4.51, br s	4′	—	—	—	3.21, m
5	—	—	—	2.80, m	5′	—	—	—	3.18, m
6	4.27, t, 9.6	4.13, t, 9.5	4.11, t, 9.6	4.14, t, 9.6	6′α	—	—	—	3.36, m
7	—	—	—	2.91, m	6′β	—	—	—	3.29, m
8*α*	—	—	—	2.23, m	1″	4.51, d, 8.0	4.47, d, 7.8	4.98, d, 7.6	4.46, d, 7.7
8*β*	—	—	—	1.35, m	2″	—	—	—	3.28, m
9*α*	—	—	—	2.11, m	3″	—	—	—	3.47, m
9*β*	—	—	—	2.41, m	4″	—	—	—	3.21, m
11	—	—	—	—	5″	—	—	—	3.28, m
13*α*	6.10, d, 3.6	6.02, d, 3.5	6.01, d, 3.6	6.02, d, 3.5	6″α	—	—	—	3.36, m
13*β*	5.57, d, 3.6	5.61, d, 3.1	5.59, d, 3.6	5.61, d, 3.5	6″β	—	—	—	3.29, m
14*α*	5.02, br s	4.91, s	5.19, br s	4.91, br s	1‴	—	4.36, d, 7.8	—	4.52, d, 7.8
14*β*	4.93, br s	4.88, s	5.08, br s	4.88, br s	2’—OH	—	—	—	5.10, d, 4.1
15*α*	5.40, br s	5.38, br s	5.36, br s	5.38, br s	2″—OH	—	—	—	5.24, d, 3.2
15β	5.35, br s	5.20, br s		5.20, br s	2‴—OH	—	—	—	5.26, d, 3.4

Note: The 1⁗, 2⁗, 3⁗, 4⁗, 5⁗, 6⁗α, and 6⁗β data of compound **63** were 4.36, d, 7.9; 3.05, m; 3.18, m; 3.04, m; 3.24, m; 3.42, m; and 3.29, m.

**Table 11:** ^13^C-NMR data of compounds **1**–**63**.

NO.	1 [6]	2 [7]	3 [8]	4 [9]	5 [10]	6 [10]	7 [11]	8 [7]	9 [12]	11 [14]	12 [7]	13 [15]	14 [16]	15 [18]	15 [17]	16 [19]	17 [15]			
	CD_3_OD	CDCl_3_	CDCl_3_	CDCl_3_	CDCl_3_	CDCl_3_	CDCl_3_	CDCl_3_	CDCl_3_	CDCl_3_	CDCl_3_	CDCl_3_	CDCl_3_	CD_3_OD	C_5_D_5_N	CDCl_3_	CDCl_3_			
1	52.7	53.1	52.1	50.6	59	47.9	46.3	52.5	47.3	47.6	41.4	42.1	42.2	43.6	43	43.3	43.5			
2	26.4	34.2	26.2	21.6	40	39.7	39.7	26	30.5	32.5	34.8	38.3	38.3	35.5	39.6	38.6	38.7			
3	40.6	30.2	39.2	40.3	27.1	27	27.3	31	32.8	30.2	73.9	78.3	78.4	79.1	78.1	73.6	73.5			
4	81.9	49.4	83.5	80	56.1	55	55.9	152	152	151.1	40.6	47	47	48.2	47.9	153.3	153.2			
5	53.4	58.2	49.1	51.2	51.5	53.3	51.5	51.2	52.2	52	47.8	50.6	52.9	52.3	51.5	49.7	49.5			
6	32.8	24.9	29.3	130.1	141.8	159.8	135	84	85.6	85.1	83.4	85.9	85.9	83.9	82.5	83.7	83.7			
7	48.6	28.9	39	131.6	132.7	142	133.2	48.6	42.3	45.1	46.3	52.9	50.6	60	59.2	39.3	50.8			
8	31.5	45.2	32	23.5	25	19.2	25.3	26.5	32.8	30.8	29.2	32.7	32.8	77.1	76.3	28.7	32.3			
9	42.6	78.5	36.6	42.7	67	27.5	27.7	40.6	37.9	36.1	39.4	37	37	48.9	48.9	36	35.9			
10	75.5	36.4	75	75	213.8	75.8	76.5	74.7	150.2	149.2	148.5	149.2	149.3	146.3	146.1	149	148.8			
11	153.5	146.6	149.5	22.8	33	32.8	33.1	42.8	50.1	139.7	39.7	42	42.1	43.5	42.9	46.3	42			
12	108.3	114.2	111.1	21.7	22.1	19.9	19.8	178.8	179	169.5	180.3	178.6	178.6	181.9	179.5	179.7	179.8			
13	20.3	22.7	66.1	—	24.7	22.1	22.1	13.2	13.5	120	11.8	13	13.1	16.8	19	11.4	13.1			
14	23.8	65	25.4	—	34.6	193	68.7	27.1	112.1	112.5	111.8	112.5	112.5	115.1	114.2	113.4	113.5			
15	26.3	180.1	32.3	—	19.9	19.8	20	107.9	109.5	109.5	8.3	14.1	18.1	18.8	17	111.4	111			
1′	—	—	173	—	—	—	171	—	—	—	—	—	—	—	—	—	—			
2′	—	—	43.6	—	—	—	21	—	—	—	—	—	—	—	—	—	—			
3′	—	—	25.8	—	—	—	—	—	—	—	—	—	—	—	—	—	—			
4′	—	—	22.6	—	—	—	—	—	—	—	—	—	—	—	—	—	—			
5′	—	—	22.6	—	—	—	—	—	—	—	—	—	—	—	—	—	—			
**NO.**	**18 [20]**	**20 [22]**	**21 [18]**	**23 [25]**	**24 [18]**	**24 [18]**	**25 [27]**	**27 [7]**	**28 [29]**	**30 [31]**	**31 [32]**	**32 [33]**	**33 [34]**	**34 [35]**	**35 [36]**	**36 [8]**	**37 [37]**			
	**CDCl_3_**	**CDCl_3_**	**CDCl_3_**	**CDCl_3_**	**CDCl_3_**	**CD_3_OD**	**C_5_D_5_N**	**CDCl_3_**	**CD_3_OD**	**CDCl_3_**	**CDCl_3_**	**CDCl_3_**	**CDCl_3_**	**CDCl_3_**	**CDCl_3_**	**CDCl_3_**	**CDCl_3_**			
1	43.3	43.6	44.2	43.9	45.2	46	44.7	40.1	39.8	40	39.6	43.7	43.7	44.1	43.8	44.6	62.9			
2	38.6	38.6	39	38.7	39.2	40	39.9	45.4	44	45.7	44.6	36.2	36.2	36.6	36.3	36.7	38.1			
3	73.6	73.7	73.6	73.2	73.7	73.1	73.1	217.6	219.2	217.7	204.4	74.4	74.4	73.8	73.7	74.4	74			
4	153.2	152.7	153	152.7	152.4	154.3	155.2	53.3	50.6	52.9	144.4	148.9	148.9	148.8	148.8	148.3	145.1			
5	49.7	49.9	50.7	45.4	51.3	51.9	50.5	45.4	47.1	44.6	48.6	50.1	50.1	50.4	50	51.2	53.1			
6	83.6	78.9	79.1	83.7	79	80.9	78.8	88.5	88.8	88.8	86.8	83.7	83.7	83.9	83.8	79.1	81.8			
7	39.3	53.4	56	49.6	51	51.7	50.1	43.7	44	44.2	44	45.7	45.7	50	45.6	55.8	55.5			
8	28.7	69.9	74.9	28.8	71.9	74.1	66	29.3	31.8	31.8	31.6	28.7	28.7	32.4	28.7	75.1	24.9			
9	36	45	44.8	32.6	41.3	42.9	44.2	38.5	38.7	38.4	38.2	36.2	36.2	36.3	36.3	45.3	37.4			
10	148.9	143.1	143.2	147.8	142.7	144.7	145.1	149.6	148.7	149.1	148.2	148.4	148.4	148.8	148.9	143.1	70			
11	46.3	38.1	42	139.6	138.1	140.6	137.7	39.3	138.7	139	138.6	39.2	39.2	42.1	39.2	42.1	42.4			
12	179.6	179.1	178.6	170.3	169.9	172	170.1	179.8	169.9	169.9	not detected	179.6	179.6	178.4	179.6	178.6	177.6			
13	11.4	11.2	15.9	120.2	123.2	122.9	120.9	11.6	121.3	121.2	121.4	11.4	11.4	13.2	11.3	16.1	12.4			
14	113.3	115.9	116.2	115.2	117.1	117	115.9	112.6	113.1	113.1	113.6	113.4	113.4	113.5	113.2	116.4	20.7			
15	111.3	112.6	112	110.9	113.2	112.1	109	70.5	14.2	68.1	122.1	113.2	113.2	112.9	113.1	114.3	118.3			
1′	—	—	—	—	—	—	—	59.3	—	66.7	—	172.8	172.8	166.3	166.2	173	172.5			
2′	—	—	—	—	—	—	—	—	—	14.9	—	43.6	43.6	116	115.9	43.8	43.6			
3′	—	—	—	—	—	—	—	—	—	—	—	25.7	25.7	157.3	157.2	25.9	25.7			
4′	—	—	—	—	—	—	—	—	—	—	—	22.4	22.4	27.4	27.4	22.6	22.3			
5′	—	—	—	—	—	—	—	—	—	—	—	22.4	22.4	20.3	20.2	22.6	22.3			
**NO.**	**38 [37]**	**39 [33]**	**41 [7]**	**42 [11]**	**43 [8]**	**44 [37]**	**45 [37]**	**46 [37]**	**47 [37]**	**48 [37]**	**49 [37]**	**50 [39]**	**51 [37]**	**52 [22]**	**52 [22]**	**53 [22]**	**54 [40]**			
	**CDCl_3_**	**CDCl_3_**	**CDCl_3_**	**CDCl_3_**	**CDCl_3_**	**CDCl_3_**	**CDCl_3_**	**CDCl_3_**	**CDCl_3_**	**CDCl_3_**	**CDCl_3_**	**CDCl_3_**	**CDCl_3_**	**CD_3_OD**	**C_5_D_5_N**	**CD_3_OD**	**CD_3_OD**			
1	43.9	44.6	43.1	44.2	45	44.2	43.5	44.3	44.1	44.1	43.5	44.3	45.9	45.8	44	46.3	45.7			
2	37.3	34.6	32.2	36.2	35.7	36.2	36	36.3	36.4	36.5	36.3	36.4	36.4	38.5	37.6	38.4	38.4			
3	73.5	74.3	73.4	74.3	74.3	74.3	74.4	74.3	74.3	74.3	74.4	75.5	74.3	81.7	80.4	81.6	81.4			
4	148.4	147.8	148.7	148.1	147.1	148.5	149	148.3	148.4	148.6	149.2	148.4	148.6	151.5	150.7	151	151.1			
5	50.4	50.2	48.8	47.8	49.7	50.3	50	50.3	50	50	50.1	50.3	49.9	52	50.2	52.5	51.4			
6	83.6	83.9	83.9	83.7	81.5	82.2	81.2	83.3	84.8	84.3	81	83	84.2	85.7	83.6	81.2	85.1			
7	49.2	45.2	44.7	50.1	165.3	52.7	55	51.9	48.9	47.9	53.4	52	46.7	41.4	39.6	54.7	45.6			
8	29.9	30.6	26.8	25	29.9	24.9	27.4	25.5	32.4	32.2	27.1	25.2	30.6	30	28.6	70.9	33.1			
9	122.2	36.6	34	34.7	30.7	34.6	36	34.4	35.9	36.1	36.1	34.6	36.4	37	36	46	36.1			
10	137	148.2	57.3	148	147.9	148.2	147.7	148.1	148.3	148.5	148	148.2	148.5	151.4	—	145.9	150.8			
11	42.2	139.5	139.3	75	125.7	75.7	76.1	76.4	46.4	43.5	77.5	76.5	43.9	47.7	45.5	40.5	49.5			
12	178.3	170	169.9	177.5	173.4	177.2	180.3	177.7	179.6	179	177.8	176	176.5	183.1	179.6	182.5	178.9			
13	12.9	120.3	121	64.3	55	41.2	40.9	42.8	38.3	37.1	38	44.4	40.8	12.2	11.4	11.8	67.6			
14	27.9	114.3	50.3	114	114.3	113.5	113.4	113.8	113.7	113.6	113.3	114	113.5	114.2	113	116.4	114			
15	116.8	113.4	112.6	114.1	118.3	114	114.4	114.2	113.4	113.2	113.9	114.5	113	114.2	112.2	115.7	113.2			
16	—	—	—	—	—	64.8	63.5	64.9	67	64.9	65.7	210.4	—	—	—	—	—			
17	—	—	—	—	—	24.9	24.4	24.9	24.3	23.9	24.6	32.3	—	—	—	—	—			
1′	173	172.8	166.2	173	173.1	173	172.9	172.9	172.8	172.8	172.8	173.1	172.8	103.3	103.8	102.5	103.2			
2′	43.6	43.6	115.9	43.6	43.8	43.6	43.6	43.6	43.6	43.6	43.6	43.8	43.6	75.7	75.3	75.8	75.3			
3′	25.8	25.8	157.9	25.8	25.9	25.7	25.7	25.8	25.8	25.8	25.7	25.9	25.8	78.7	78.6	78.8	78.2			
4′	22.4	22.4	27.6	22.4	22.6	22.4	22.4	22.4	22.4	22.4	22.4	22.5	22.4	72.2	71.7	72.4	71.8			
5′	22.4	22.4	20.5	22.4	22.6	22.4	22.4	22.4	22.4	22.4	22.4	22.6	22.4	78.4	78.4	78.4	77.9			
6′	—	—	—	—	—	—	—	—	—	—	—	—	—	63.3	62.9	63.4	62.9			
**NO.**	**55 a [41]**	**56 b [42]**	**57 c [12]**	**57 a [22]**	**57 d [22]**	**58 e [43]**	**59 a [44]**	**60 e [45]**	**61 b [46]**	**61 e [25]**	**62 b [46]**	**62 b [25]**	**63 b [46]**	**NO.**	**58 e [43]**	**59 a [44]**	**60 e [45]**	**62 b [46]**	**62 b [25]**	**63 b [46]**
1	44.4	42.1	45.4	44.5	43.7	46.2	45.9	46.1	43.5	46.2	43.4	43.4	43.9	1″	130.9	126.7	127.8	103.9	103.9	103.9
2	37.9	37.3	38	38	37.3	38.6	37.3	37.8	36.9	38.5	36.9	36.9	37.4	2″	129	114.9	115.4	72.4	72.4	73.4
3	80.5	86.2	80.7	80.5	79.7	81.3	79.5	80.9	83.3	81.4	83.2	83.2	79.8	3″	130	148.2	149.7	73.8	73.8	86.6
4	150.8	44.1	150.2	150.6	150.3	150.8	150.2	150.1	150	150.6	149.9	150	150.4	4″	150.1	147.3	146.8	79.4	79.4	68.9
5	50.8	49.5	50.7	50.1	49.1	51.8	51.6	52.4	48.8	51.7	48.8	48.8	49.2	5″	130	116.3	116.5	76.1	76.1	73.2
6	79.4	86.2	84.7	83.6	83.6	85.2	83.1	80.8	76.9	85.2	86.6	86.6	83.7	6″	129	122.1	123	60.8	61.1	63.5
7	56.3	46.8	45.8	45.1	44.6	46.4	45.9	54.4	44.3	46.5	44.3	44.3	44.7	1‴	172.3	166.4	168.3	103.4	103.4	103.5
8	75.1	30.6	31	30.6	30.4	31.6	30.4	70.6	30.1	31.6	30.1	30.1	30.5	2‴	121.7	115.6	115.3	72.7	72.7	73
9	46.6	35.7	33.9	34.2	34.1	34.4	33.8	46.4	33.8	34.4	33.8	33.7	34.2	3‴	144.6	145.9	147.3	76.9	76.9	87.2
10	145.1	149.5	149.3	148.9	149.1	150	149	144.8	148.7	150	148.7	148.8	149.2	4‴	—	—	—	68.5	68.5	69
11	42.3	140.1	141.5	141	140.5	142.1	140.5	39.9	140.2	142.1	140.1	140.1	140.6	5‴	—	—	—	76.3	76.1	76.6
12	179	169.6	171.6	170	169.9	172.3	169.9	181.9	169.6	172.2	169.5	169.5	170	6‴	—	—	—	61.1	61	61.3
13	16.5	119.5	120	119.4	120.1	120.4	119.5	11.5	119.8	120.4	119.7	119.9	120.2		—	—	—	—	—	—
14	115.1	112.7	114.1	113.9	113.9	114.7	114.9	116.6	113.6	114.7	113.6	113.6	114	NO.	**61 b** [46]	**61 e** [25]	—	—	—	—
15	112.3	18.1	112.8	112.1	111	113.6	114.9	116.2	110.8	113.8	110.8	110.8	111.1	1″	104.1	105.3	—	—	—	—
1′	104	104.1	102.3	104.3	102.9	103.1	98.5	99.4	101.7	102.3	101.7	101.7	102.2	2″	72.3	75.6	—	—	—	—
2′	75.3	73.6	74.3	75.3	73.9	78.2	76.1	75.3	70.2	74.6	70.1	70.1	72.8	3″	73.9	78.3	—	—	—	—
3′	78.2	76.8	77.5	78.6	77.2	75.3	74.9	76.4	88.3	88.3	88	88	88.4	4″	79.4	71.5	—	—	—	—
4′	71.8	70.2	71.1	71.7	70.5	71.8	71.8	72.1	68.5	70.2	68.4	68.4	68.9	5″	76.1	77.8	—	—	—	—
5′	78.5	76.8	77.2	78.5	77.2	77.9	78.2	78.1	76.3	77.5	76.3	76.3	76.8	6″	61.1	62.7				
6′	62.9	61.2	62.1	62.8	61.5	62.8	62.6	62.8	60.9	62.6	60.8	60.9	63.5							

Note: The 1″, 2″, 3″, and 4″ data of compound **51** were 176.3; 32.4; 18.3; and 48.8, respectively. The ^13^C-NMR data of 1″ and 2″ of **54** were recorded as 67.8 and 15.3. a: Measured in C_5_D_5_N. b: Measured in DMSO. c: Measured in CDCl_3_. d: Measured in CD_3_SOCD_3_. e: Measured in CD_3_OD.

**Table 12:** The compound name, molecular formula, and NMR test reagent of germacrane-type sesquiterpenes.

No.	Compound Name	Molecular Formula	Solvent	Ref.
**64**	Isodihydrocostunolide	C_15_H_22_O_2_	CDCl_3_	[48]
**65**	Taraxinic acid	C_15_H_18_O_4_	CDCl_3_	[49]
**66**	Yunnanolide K	C_15_H_20_O_4_	CDCl_3_	[11]
**67**	Germacra-1(10), 4-diene-11*α*-methyl-12,8*α*-olide-15-acid	C_15_H_20_O_4_	DMSO/CD_3_OD	[34,50]
**68**	Germacra-1(10),4,11(13)-triene-12,8*α*-olide-15-acid	C_15_H_18_O_4_	CD_3_OD	[50]
**69**	Ainsliaside B	C_21_H_28_O_9_	C_5_D_5_N CD_3_OD	[44]
**70**	Taraxinsaure-1′-O-*β*-D-glucopyranoside	C_21_H_28_O_9_	CD_3_OD	[51]
**71**	Picriside B	C_21_H_30_O_8_	C_5_D_5_N	[27]
**72**	Ainsliaolide C	C_26_H_40_O_12_	DMSO	[31]
**73**	Taraxic acid-1′-O-*β*-D-glucopyranoside	C_21_H_28_O_9_	CD_3_OD/C_5_D_5_N	[2]
**74**	Germacra-1(10),4,11(13)-triene-12,8*α*-olide-15-oic acid(15-1′)-*β*-D-glucopyransyl ester	C_21_H_28_O_9_	CD_3_OD	[50]
**75**	Ainsliaea latifolia A	C_21_H_30_O_9_	CD_3_OD	[52]

**Table 13:** ^1^H-NMR data of compounds **64**–**75**.

NO.	64 [48]	65 [49]	66 [11]	67 [34]	67 [50]	68 [50]	69 [44]	71 [27]	72 [31]	73 [2]	74 [50]	75 [52]
	CDCl_3_	CDCl_3_	CDCl_3_	DMSO	CD_3_OD	CD_3_OD	C_5_D_5_N	C_5_D_5_N	DMSO	C_5_D_5_N	CD_3_OD	CD_3_OD
1	1.68–1.40, m	5.68, dd, 13.0, 3.7	5.09, dd, 12.0, 5.0	5.06, dd, 11.7, 4.5	5.06, dd, 11.7, 4.5	5.06, dd, 11.7, 4.5	6.72, br t, 7	—	4.81, m	5.64, dd, 11.0, 4.0	5.13, m	5.16, dd, 11.8, 4.7
2*α*	1.68–1.40, m	3.38, m	2.31, m	2.16, t, 12.3, 4.9	2.13, m	2.13, m	—	—	2.17, m	2.14, m	—	2.38–2.42, m
2*β*			2.18, m	2.02–2.06, m	2.02, m	2.02, m	—	—	2.26, m	3.54, m	—	2.14–2.20, m
3*α*	2.10, d, 2.7	2.25, m	2.93, m	2.66–2.72, overlapped	2.69, m	2.69, m	—	—	1.83, m	2.3–2.0, m	—	2.92, dd, 12.5, 3.9
3*β*	1.9–1.7, m	2.35, m	1.88, m	1.78, t, 12.3, 5.2	1.78, m	1.78, m	—	—	2.61, m		—	1.90–1.98, m
5	5.15, d, 6	4.91, d, 10.0	5.51, dd, 11.0, 2.5	5.38, dd, 11.1, 2.1	5.38, dd, 11.1, 2.1	5.38, dd, 11.1, 2.1	4.81, d, 10	—	4.75, br s	4.94, dd, 10.0, 1.2	5.74, m	5.65, dd, 11.3, 2.6
6*α*	—	—	3.13, m	3.01, t, 16.1, 10.8	3.01, m	3.01, m	4.58, dd, 10, 9	—	—	—	—	3.19–3.24, m
6*β*	4.8, m	4.58, dd, 10.0, 9.0	2.49, d, 16.5	2.41–2.55, overlapped	2.44, m	2.44, m	—	—	4.77, d, 9.6	4.72, dd, 10.0, 10.0	—	2.55–2.65, m
7	3.6, m	2.56, m	2.31, m	1.82–1.90, m	1.86, m	1.86, m	—	—	1.63, m	2.54, m	—	1.92–2.01, m
8*α*	1.68–1.40, m	2.19, m	4.39, t, 11.5	—	—	—	—	—	1.72, m	2.3–2.0, m	—	—
8*β*			—	4.19–4.25, m	4.22, m	4.22, m	—	—	2.06, m		4.27, m	4.27–4.37, m
9*α*	1.68–1.40, m	2.90, m	2.93, m	2.66–2.72, overlapped	2.66, m	2.66, m	—	—	2.24, m	2.86, m	—	2.83, d, 12.3
9*β*		2.15, m	2.28, m	2.27, t, 11.8	2.27, m	2.27, m	—	—	1.96, m	2.3–2.0, m	—	2.33–2.38, m
10	2.5, m	—	—	—	—	—	—	—	—	—	—	—
11	—	—	2.78, m	2.41–2.55, overlapped	2.51, m	—	—	—	2.33, m	—	—	2.54–2.58, m
13*α*	6.2, d, 2	6.24, d, 3.4	1.26, d, 7.5	1.11, d, 7.0	1.11, d, 7.0	6.08, m	—	5.51, d, 3.2	1.09, d, 6.6	6.23, d, 3.5	6.23, dd, 18.8, 3.0	1.28, d, 7.0
13*β*	5.65, d, 2	5.51, d, 2.9				5.79, m	—	6.35, d, 3.6			5.74, m	
14	1.1, d, 8	—	1.35, s	1.27, s	1.27, s	1.31, s	—	1.37, br s	1.30, s	—	1.41, s	1.41, s
15*α*	1.2, s	1.60, s	—	—	—	—	1.71, br s	—	3.78, br s	1.72, d, 1.2	—	—
15*β*			—	—	—	—		—	4.40, br s		—	—
1′	—	—	—	—	—	—	6.26, d, 8	4.96, d, 7.5	4.17, d, 7.8	6.18, d, 7.6	5.52, d, 7.9	5.56, d, 7.8
2′	—	—	—	—	—	—	—	—	2.97, m	4.44–3.80, m		3.43, overlapped
3′	—	—	—	—	—	—	—	—	3.13, m	4.44–3.80, m		3.43, overlapped
4′	—	—	—	—	—	—	—	—	2.99, m	4.44–3.80, m		3.43, overlapped
5′	—	—	—	—	—	—	—	—	3.23, m	4.44–3.80, m		3.43, overlapped
6′*α*	—	—	—	—	—	—	—	—	3.44, d, 11.2, 7.2	4.44–3.80, m	3.81, m	3.84, dd, 12.0, 2.0
6′β	—	—	—	—	—	—	—	—	3.81, d, 11.2, 1.8	4.44–3.80, m	3.69, m	3.75, dd, 12.0, 4.4

Note: The ^1^H-NMR data of 1″, 2″, 4″*α*, and 4″*β* and 5″ of **72** were recorded as 4.88, br s; 3.74, m; 3.57, d, 9.6; 3.83, d, 9.6; and 3.33, s, respectively.

**Table 14:** ^13^C-NMR data of compounds **64**–**75**.

NO.	64 [48]	65 [49]	66 [11]	67 [34]	67 [50]	68 [50]	69 [44]	70 [51]	71 [27]	72 [31]	73 [2]	74 [50]	75 [52]
	CDCl_3_	CDCl_3_	CDCl_3_	DMSO	CD_3_OD	CD_3_OD	CD_3_OD	CD_3_OD	C_5_D_5_N	DMSO	CD_3_OD	CD_3_OD	CD_3_OD
1	16.7	149.9	128.8	128.9	128.9	130.4	143	149.7	126.9	125.9	149.6	130	129.8
2	32.6	26.7	26.8	27.1	27.1	27.6	26.2	27.6	27.7	25.9	27.6	27.7	27.9
3	42.6	39.2	34.8	34.9	34.9	35.7	38	39.9	36	34.9	40	35.5	35.7
4	148.9	139.8	124.1	125.1	125.1	126.6	141.3	141.8	141	138.8	141.8	126.6	125.5
5	118.5	126	150.7	148.6	148.6	149.2	127.1	127.2	130.1	130.1	127.1	151.6	152
6	76.4	82.1	28.6	31.1	31.1	32.8	82.8	83.8	80.3	78.7	83.8	32.8	32.4
7	39.5	50.3	48.3	52.6	52.6	50.1	46.7	51.1	50.8	53.7	51.1	50.1	54.4
8	41.7	30.1	82.4	82.6	82.6	85.1	24.2	31.3	27.1	27	31.3	85	84.5
9	32.7	36.7	46.2	45.5	45.5	47.2	26.4	37.2	41.1	40.7	37.2	47.2	46.8
10	37.5	130.3	134.2	134	134	134.3	135.1	131.9	137.6	137	131.9	135.1	135.6
11	139.9	143.2	40.7	41.4	41.4	141.2	139	144.5	141.2	41	144.4	141.1	43
12	170.4	170.6	178.7	178	178	171.7	172.4	172.7	170.3	178.1	172.6	171.7	180.5
13	121.5	120.1	11.7	13.1	13.1	121.2	119.4	120.4	119.1	12.8	120.4	121.2	13.2
14	22.5	173.3	16.8	16.8	16.8	17.1	167.6	169.8	16.3	15.8	167.8	17.4	17.3
15	28.6	17	171.9	169	169	167.5	17.2	17.3	67.8	65.8	17.3	167.5	167.7
1′	—	—	—	—	—	—	95.7	95.3	105.3	103.1	95.3	95.7	95.7
2′	—	—	—	—	—	—	73.9	73.9	75.2	73.3	73.9	74	74
3′	—	—	—	—	—	—	78.6	78.3	78.6	76.6	78.3	78.7	78.4
4′	—	—	—	—	—	—	71	71	71.8	70.2	71	71	71
5′	—	—	—	—	—	—	77.9	78.7	78.6	75.8	78.7	78.4	78.7
6′	—	—	—	—	—	—	62.4	62.3	62.9	67.5	62.4	62.2	62.2

Note: The ^13^C NMR of 1″, 2″, 3″, 4″, and 5″ of **72** were 109.3; 75.6; 78.7; 73.2; and 63.3, respectively.

**Table 15:** The compound name, molecular formula, and NMR test reagent of eudesmane sesquiterpenes.

No.	Compound Name	Molecular Formula	Solvent	Ref.
**76**	1*β*-Hydroperoxygermacra-4(15),5,10(14)-triene	C_15_H_24_O_2_	CDCl_3_	[12]
**77**	Selin-11-en-4α-ol	C_15_H_26_O	CDCl_3_	[54]
**78**	4*α*-Hydroxy-4*β*-methyldihydrocostol	C_15_H_26_O_2_	CDCl_3_	[55]
**79**	Cyperusol C	C_15_H_26_O_2_	CDCl_3_	[56]
**80**	1*β*,4*β*-Dihydroxyeudesman-11-ene	C_15_H_26_O_2_	CDCl_3_	[57]
**81**	*α*-Dictyopterol	C_15_H_24_O	CDCl_3_	[58]
**82**	1*β*,6*α*-Dihydroxy-4(15)-eudesmane	C_15_H_26_O_3_	CDCl_3_	[59]
**83**	1-oxo-5*α*,7*α*H-eudesma-3-en-15-al	C_15_H_22_O_2_	CDCl_3_	[60]
**84**	1*β*-Hydroxy-*α*-cyperone	C_15_H_22_O_2_	CDCl_3_	[61]
**85**	(-)-*α*-Cadinol	C_15_H_26_O	CDCl_3_	[62,63]
**86**	T-Cadinol	C_15_H_26_O	CDCl_3_	[63,64]
**87**	10-Hydroxyl-15-oxo-*α*-cadinol	C_15_H_24_O_2_	CDCl_3_	[65,66]
**88**	15-oxo-T-cadinol	C_15_H_24_O_2_	CDCl_3_	[66]
**89**	Ainsliaea acid B	C_15_H_18_O_4_	CD_3_OD	[67]
**90**	4-Acrylic-6-methyl-*α*-tetralone	C_14_H_14_O_3_	CDCl_3_	[67]
**91**	4*α*-Hydroxy-12-acetoxy-eudesm-11(13)-en	C_17_H_28_O_3_	—	[68]
**92**	4*α*-Hydroxy-eudesm-11-en-12-isovaleroxyl	C_20_H_34_O_3_	CDCl_3_	[34]
**93**	Ainsliatone A acid	C_14_H_20_O_5_	CD_3_OD	[42]
**94**	Ainsliatone B	C_15_H_22_O_5_	CDCl_3_	[31]
**95**	Ainslide B	C_17_H_26_O_3_	CDCl_3_	[7]
**96**	Spicatene C	C_20_H_32_O_3_	CDCl_3_	[8]
**97**	6,11-Diacetoxy-1,4-dihydroxyeudesmane	C_19_H_32_O_6_	CD_3_OD	[40]
**98**	Alatoside N	C_20_H_34_O_6_	CD_3_OD	[42]
**99**	Alatoside M	C_21_H_32_O_8_	CD_3_OD	[42]
**100**	Ainsliaside C	C_21_H_36_O_8_	C_5_D_5_N	[69]
**101**	Ainsliaside D	C_21_H_36_O_8_	C_5_D_5_N	[69]
**102**	Ainsliaside E	C_21_H_38_O_9_	C_5_D_5_N	[69]
**103**	Alantolactone	C_15_H_20_O_2_	CDCl_3_	[70]
**104**	Isoalantolactone	C_15_H_20_O_2_	CDCl_3_	[70]
**105**	Pertyolides B	C_17_H_24_O_4_	CDCl_3_	[39]
**106**	Pertyolides A	C_17_H_24_O_4_	CDCl_3_	[39]
**107**	Ainsliatone A	C_14_H_18_O_4_	CDCl_3_	[71]
**108**	4(15)-En-eudesma-6,12-olide-15-O-*β*-D-glucopyranoside	C_21_H_32_O_8_	CD_3_OD	[40]
**109**	Ixerin W	C_22_H_30_O_7_	C_5_D_5_N	[27]
**110**	3(4)-En-eudesma-6,12-olide-15-O-*β*-D-glucopyranoside-O-*β*-D-glucopyranoside	C_21_H_32_O_8_	CD_3_OD	[40]

**Table 16:** ^1^H-NMR data of compounds **76**–**84**.

NO.	76 [12]	77 [54]	78 [55]	79 [56]	80 [57]	81 [58]	82 [59]	83 [60]	84 [61]
	CDCl_3_	CDCl_3_	CDCl_3_	CDCl_3_	CDCl_3_	CDCl_3_	CDCl_3_	CDCl_3_	CDCl_3_
1	4.15, dd, 12.0, 3.5	—	—	3.32, dd, 11.2, 4.4	3.27, dd, 11.4, 4.8	3.49–3.56, m	3.43, dd, 11.7, 4.6	—	3.83, dd, 13, 5.5
2*α*	2.05, m	—	—	1.62, m	1.96, m	1.45–1.55, m	1.86, m	2.54, m	2.64, dd, 16.5, 5.5
2*β*		—	—	1.72, m	1.91, m		1.55, m		2.56, dd, 16.5, 13
3*α*	2.27, ddd, 2.5, 5.5, 13.0	—	—	1.52, ddd, 13.5, 12.0, 3.5	1.16, m	5.26, s	2.07, m	6.64, br d, 5.2	—
3*β*	2.46, td, 13.0, 5.0	—	—	1.79, ddd, 12.0, 3.5, 3.0	1.09, m	—	2.33, m	—	—
5	6.04, d, 16.0	1.81, dddd	—	1.28, m	1.24, dd, 9.6, 3.6	1.10–1.38, m	1.75, m	2.21, dd, 10.8, 4.8	—
6*α*	5.46, dd,16.0, 10.5	—	—	1.26, m	1.27, m	1.10–1.38, m		1.44, m	2.19, ddd
6*β*		—	—	1.84, m	1.22, m		3.72, dd, 9.8, 9.8	1.87, m	2.08, ddd, 14, 12, 1.5
7	1.5–1.86, 2.62, m	1.96, dddd, br	—	1.94, m	1.58, m	1.84–1.92, m	1.28, m	1.81, m	2.02, br dddd, 13.5, 12, 3.5, 2.5
8*α*	1.5–1.86, 2.62, m	—	—	1.61, m	1.78, m	1.84–1.92, m	1.53, m	1.58, m	1.75, m
8*β*		—	—	1.38, dddd, 17.0, 13.5, 13.0, 3.5	1.71, m		1.21, m		1.59, dddd, 13.5, 13.5, 13.5, 3.5
9*α*	1.5–1.86, 2.62, m	—	—	1.13, ddd, 13.5, 13.0, 4.0	1.88, m	1.84–1.92, m	1.17, m	2.43, ddd, 14.4, 10.8, 3.6	1.35, ddd, 13.5, 13.5, 4
9*β*		—	—	1.90, ddd, 13.5, 3.5, 3.5	1.86, m		1.92, m	2.86, ddd, 14.4, 5.4, 5.4	2.16, ddd, 13.5, 3.5, 3
11	1.5–1.86, 2.62, m	—	—	—	—	—	2.24, m	1.67, m	—
12*α*	0.83, d, 6.5	—	4.12, s	4.72, m	4.74, br s	4.71, s	0.95, d, 6.9	0.94, d, 6.8	4.78, m
12*β*		—			4.71, br s				
13*α*	0.92, d, 6.5	1.75, s	5.00, d, 1.4	0.89, s	1.76, s	1.73, s	0.87, d, 7.1	0.94, d, 6.8	1.78, dd
13*β*			4.90, d, 1.0						
14*α*	5.21, br s	1.12, s	0.88, s	1.75, s	1.05, s	1.58, s	0.71, s	1.33, s	1.18, s
14*β*	5.34, br s								
15*α*	4.89, br s	0.89, s	1.09, s	1.11, s	1.16, s	0.76, s	5.02, s	9.35, s	1.73, d, 1
15*β*	4.97, br s						4.74, s		

**Table 17:** ^1^H-NMR data of compounds **85**–**92**.

NO.	85 [63]	86 [63]	87 [66]	88 [66]	89 [67]	90 [67]	91 [68]	92 [34]
	CDCl_3_	CDCl_3_	CDCl_3_	CDCl_3_	CD_3_OD	CDCl_3_		CDCl_3_
1*α*	—	—	2.16, m	2.24, m	2.34, m	7.97, d, 8.0	—	1.36–1.46, overlapped
1*β*	—	—	1.21, m	1.21, m	1.69, m		—	1.12, m
2*α*	—	—	2.06, m	2.06, m	1.65, m	7.17, d, 8.0	—	1.53–1.62, overlapped
2*β*	—	—	2.46, m	2.45, m	1.80, m		—	
3*α*	—	—	—	—	—	—	—	1.77–1.81, m
3*β*	—	—	—	—	—	—	—	1.36–1.46, overlapped
4	5.29, br s	5.42, br s	6.87, s	6.94, s	4.99, br s	6.96, br s	—	—
5	—	—	2.01, m	2.04, m	2.75, br s	—	—	1.21–1.29, overlapped
6*α*	—	—	1.20, m	1.20, m	2.88, d, 12.0	4.25, br s	—	1.89–1.94, m
6*β*	—	—	—	—	—	—	—	1.21–1.29, overlapped
7*α*	—	—	1.72, m	1.55, m	2.26, m	2.33, m	—	2.02–2.06, m
7*β*	—	—	1.23, m	1.27, m	1.99, m	2.29, m	—	—
8*α*	—	—	1.45, m	1.47, m	6.90, m	2.56, m	—	1.53–1.62, overlapped
8*β*	—	—	1.84, m	1.78, m	—	2.64, m	—	1.36–1.46, overlapped
9*α*	—	—	—	—	—	—	—	1.21–1.29, overlapped
9*β*	—	—	—	—	—	—	—	
10	—	—	1.36, m	1.35, m	2.96, br s	—	—	—
11	1.66, br s	1.61, br s	2.21, m	2.35, m	—	—	—	—
12*α*	—	—	0.85, d, 6.8	0.87, d, 6.9	6.34, br s	6.51, br s	—	4.55–4.63, m
12*β*	—	—			5.54, br s	5.23, br s	—	
13*α*	0.91, d, 7.2	0.90, d, 6.9	0.98, d, 6.8	0.97, d, 6.9	—	—	5.03	5.03, d, 1.4
13*β*					—	—	4.6	4.98, s
14	0.78, d, 7.2	0.78, d, 6.9	1.14, s	1.24, s	—	2.36, s	0.90, s	0.89, s
15	1.05, s	1.15, s	9.43, s	9.43, s	1.60, s	—	1.09, s	1.10, s
1′	—	—	—	—	—	—	—	—
2′	—	—	—	—	—	—	2.06, s	2.23, d, 7.2
3′	—	—	—	—	—	—	—	2.10–2.17, m
4′	—	—	—	—	—	—	—	0.96, d, 6.6
5′	—	—	—	—	—	—	—	0.96, d, 6.6

**Table 18:** ^1^H-NMR data of compounds **93**–**100**.

NO.	93 [42]	94 [31]	95 [7]	96 [8]	97 [40]	98 [42]	99 [42]	100 [69]
	CD_3_OD	CDCl_3_	CDCl_3_	CDCl_3_	CD_3_OD	CD_3_OD	CD_3_OD	C_5_D_5_N
1*α*	3.80, dd, 10.0, 5.0	3.86, dd, 11.7, 5.1	3.43, dd, 11.6, 4.6	3.43, dd, 11.6, 4.6	3.12, dd, 11.0, 3.4	—	—	3.58, dd, 11, 4
1*β*	—	—	—	—	—	—	—	—
2*α*	2.08, m	1.89, m	1.78–1.87, m	1.57, m	1.46, m	5.30, br s	5.31, br s	—
2*β*	1.84, m	2.14, m	1.55–1.60, m	1.82, m	1.93, m	—	—	—
3*α*	2.21, m	2.42, m	2.32, ddd, 13.6, 4.9, 2.2	2.10, m	1.49, m	2.00, m	2.03, m	—
3*β*	2.53, m		2.06–2.17, m	2.32, m	1.58, m	2.40, m	2.41, m	—
4	—	—	—	—	—	3.67, dd, 10.0, 6.5	3.72, dd, 10.0, 6.5	—
5	1.64, m	2.24, d, 9.9	1.75–1.82, m	1.77, m	0.96, br s	—	—	2.84, d, 6
6*α*	—	—	1.67–1.74, m	1.25, m	5.78, s	1.25, m	1.26, m	4.92, br t, 5.5
6*β*	4.14, t, 10.0	4.22, dd, 10.2, 9.9	1.39–1.46, m	1.67, m	—	2.37, m	2.40, m	—
7	2.41, m	2.54, m	1.98–2.08, m	2.00, m	2.11, d, 13.6	1.95, m	2.02, m	—
8*α*	1.64, m	1.67, m	1.64–1.71, m	1.35, m	1.56, m	1.57, m	1.50, m	—
8*β*		1.74, m	1.33–1.40, m	1.69, m	1.87, m	1.72, m	1.67, m	—
9*α*	1.36, m	1.40, m	1.97, t, 3.3	1.21, m	1.09, m	1.25, m	1.26, m	—
9*β*	1.81, m	1.87, m	1.17–1.27, m	1.98, m	1.96, m	1.72, m	1.84, m	—
10	—	—	—	—	—	2.02, m	2.04, m	—
11	—	—	—	—	—	1.13, d, 7.0	—	—
12	—	—	4.59, s	4.59, s	1.41, s	1.58, s	—	1.57, s
13*α*	6.23, s	6.27, br s	5.07, d, 0.9	5.10, br s	1.45, s	0.81, s	5.58, s	1.58, s
13*β*	5.67, s	5.69, br s	5.02, s	5.00, br s			6.14, s	
14	0.76, s	0.88, s	0.71, s	0.71, s	1.31, s	—	1.58, s	1.08, s
15*α*	—	—	4.77, d, 1.5	4.50, br s	1.28, s	—	0.85, s	5.03, br s
15*β*	—	—	4.50, d, 1.5	4.77, br s		—		5.17, br s
1′	—	3.76, s	—	—	—	4.30, d, 7.5	4.30, d, 8.0	5.09, d, 8
2′	—	—	2.10, s	2.23, m	1.98, s	3.14, m	3.14, m	3.96, t, 8.5
3′	—	—	—	2.13, m	—	3.31, m	3.30, m	4.20, t, 9
4′	—	—	—	0.97, d, 6.6	—	3.27, m	3.27, m	4.24, t, 9
5′	—	—	—	0.97, d, 6.6	—	3.22, m	3.25, m	3.77, m
6′*α*	—	—	—	—	—	3.67, dd, 12.0, 6.5	3.65, dd, 12.0, 6.5	4.34, dd, 12, 4
6′β	—	—	—	—	—	3.85, dd, 11.5, 2.0	3.85, dd, 11.5, 2.0	4.37, dd, 12, 2

Note: The ^1^H-NMR data of 2″ of compound **97** were 1.98, s.

**Table 19:** ^1^H-NMR data of compounds **101**–**110**.

NO.	101 [69]	102 [69]	103 [70]	104 [70]	105 [39]	106 [39]	107 [71]	108 [40]	109 [27]	110 [40]
	C_5_D_5_N	C_5_D_5_N	CDCl_3_	CDCl_3_	CDCl_3_	CDCl_3_	CDCl_3_	CD_3_OD	C_5_D_5_N	CD_3_OD
1*α*	3.78, dd, 8, 7	3.63, dd, 10, 5	1.40–1.82, m	1.50–2.20, m	1.15, m	1.23, m	3.96, dd, 11.0, 4.8	1.38, m	5.46, dd, 10, 2	1.52, m
1*β*	—	—			1.62, m	1.53, m	—	1.45, m	—	1.44, dd, 13.1, 7.0
2*α*	—	—	1.40–1.82, m	1.50–2.20, m	1.40, m	1.26, m	1.91, m	1.55, m	5.86, dd, 10, 3	2.15, m
2*β*	—	—			1.80, m	1.60, m	2.21, m			2.20, m
3*α*	5.43, br s	—	1.40–1.82, m	1.50–2.20, m	1.56, m	2.00, m	2.40, m	1.53, m	6.36, br s	5.81, s
3*β*	—	—				2.34, m	2.51, m	3.00, d, 11.7	6.36, br s	5.81, s
4	—	—	2.43, m	—	2.46, m	—	—	—	—	—
5	3.25, br s	2.66, d, 3	—	2.34, m	—	1.81, d, 12.4	2.62, d, 11.0	2.08, d, 11.0	2.55, br d, 11	2.33, d, 10.5
6*α*	4.91, t, 3	5.09, t, 3	5.13, d, 8	1.71, m	4.94, d, 3.5	1.06, q, 12.4	—	4.06, t, 11.0	4.22, overlapped	4.25, t, 10.5
6*β*	—	—		1.24, m		1.42, m	4.11, t, 11.0			
7	—	—	3.56, m	2.97, m	3.01, dd, 5.4, 3.5	2.38, m	2.45, m	1.65, overlapped	2.50, m	1.61, m
8*α*	—	—	4.80, m	4.48, m	5.13, dt, 5.4, 3.0	5.04, m	1.59, m	1.79, m	—	1.81, d, 11.7
8*β*	—	—					2.10, m	1.58, m	—	1.63, m
9*α*	—	—	2.09, dd, 6, 6	1.99, m	2.14, dd, 14.9, 3.0	1.46, dd, 15.6, 4.5	1.50, m	1.55, m	—	1.36, d, 11.2
9*β*	—	—	1.53, m	1.39, m	1.51, dd, 14.9, 3.0	2.20, dd, 15.6, 2.6	2.06, m	1.35, m	—	1.58, m
11	—	—	—	—	—	—	—	2.43, dq, 10.6, 6.9	—	2.37, m
12	1.49, s	1.52, s	—	—	—	—	—	—	—	—
13*α*	1.61, s	1.63, s	6.17, d, 4	6.11, d, 2	2.65, d, 17.5	2.64, d, 17.5	5.41, d, 3.0	1.15, d, 6.9	5.36, d, 3.1	1.16, d, 6.8
13*β*			5.60, d, 4	5.57, d, 2	2.95, d, 17.5	3.02, d, 17.5	6.09, d, 3.0		6.15, d, 3.2	
14	1.20, s	1.33, s	1.17, s	0.82, s	1.22, s	0.78, s	0.86, s	0.88, s	0.92, s	0.97, s
15*α*	2.15, br s	1.47, s	1.07, s	4.76, d, 3	1.13, d, 7.7	4.79, s	—	6.16, br s	5.18, br s	4.38, d, 11.7
15*β*				4.43, d, 3		4.43, s	—		5.13, br s	4.15, d, 11.7
17	—	—	—	—	2.33, s	2.34, s	—	—	—	—
1′	5.15, d, 8	5.30, d, 8	—	—	—	—	—	4.48, d, 7.7	5.06, d, 7	4.39, d, 7.7
2′	4.01, t, 8.5	4.01, t, 8.5	—	—	—	—	—	3.28, m	—	3.17, m
3′	4.25, t, 8.5	4.19, t, 9	—	—	—	—	—	3.35, m	—	3.33, m
4′	4.20, t, 9	3.94, t, 9	—	—	—	—	—	3.35, m	—	3.28, m
5′	3.92, m	4.06, dt, 8, 2	—	—	—	—	—	3.28, m	—	3.25, m
6′*α*	4.31, dd, 12, 5	4.16, dd, 11, 8.5	—	—	—	—	—	3.84, dd, 12.1, 2.3	—	3.86, dd, 11.9, 2.0
6′*β*	4.42, dd, 12, 2	4.67, dd, 11, 1.5	—	—	—	—	—	3.69, dd, 12.1, 5.0	—	3.67, dd, 11.9, 5.1

**Table 20:** ^13^C-NMR data of compounds **76**–**110**.

NO.	76 [12]	77 [54]	78 [55]	79 [56]	80 [57]	82 [59]	83 [60]	84 [61]	85 [62]	86 [64]	87 [65]	88 [66]	89 [67]	90 [67]	92 [34]	93 [42]	94 [31]
	CDCl_3_	CDCl_3_	CDCl_3_	CDCl_3_	CDCl_3_	CDCl_3_	CDCl_3_	CDCl_3_	CDCl_3_	CDCl_3_	CDCl_3_	CDCl_3_	CD_3_OD	CDCl_3_	CDCl_3_	CD_3_OD	CDCl_3_
1	89.9	43.3	42.2	79.3	79.7	79.1	212.2	74.4	21.9	22.6	21.3	21.2	26.5	127.5	41	77.3	77.2
2	29.3	20.1	20.1	28.5	25.6	31.9	38.9	42.4	30.9	30.9	22.2	22	27.4	128.6	20.1	31.6	30
3	30.7	44.6	43.5	40.8	39.5	35.1	158.6	197.5	135	134.3	142.4	141.1	138.2	145.1	43.4	40.6	39.2
4	146.4	72.2	72.2	71.6	71.3	146.1	143.7	129.5	122.3	122.6	151.6	152.8	119.6	129.6	72.1	212.6	212.1
5	129.6	54.9	55	52.9	50.4	55.9	53.1	161.9	39.8	37.7	41.4	39.3	38	142	54.9	62.2	61.3
6	138.1	26	27.3	25.7	26.4	67	25	32.8	46.7	46.6	45.6	45.6	38.9	40.4	26.2	67.6	67.5
7	52.7	46.3	41.1	45.7	46.1	49.3	55.8	45.1	22.7	19.8	22.1	26.4	26.6	27.5	42.6	48.4	46
8	35.6	26.8	26.6	26.4	26.8	18.1	26.8	26.5	42.2	40.3	41.8	40	140.8	35	27.1	28.1	26.6
9	36.5	41	44.7	40.5	39.3	36.3	35.1	37.7	72.4	70.7	72.1	70.6	134	198.1	44.6	37.5	36
10	148	34.6	34.7	38.9	38.9	41.7	59.6	41.3	50	47.9	49.6	47.6	36.1	130.5	34.6	45.7	43.8
11	31.9	150.7	154.1	150.3	150.6	26	32.3	148.9	25.9	26.1	26.2	28.6	143.8	144.7	148.8	125.9	141.9
12	20.5	108.1	65.3	108.3	108.6	21.1	19.4	109.4	21.1	21.4	21.4	21.2	124.9	130.7	65.8	170.5	167.6
13	20.7	22.7	107.9	21	20.7	16.2	21.9	20.6	15.1	15.2	15.2	15.2	170.5	171.3	110.7	144.1	125
14	114.6	18.6	18.7	13	12.5	11.6	19.6	16.3	20.8	28.4	20.5	19.9	171.5	21.9	18.7	12.2	11.9
15	113.2	21	22.7	22.7	29.7	107.8	192.7	11	23.8	23.7	194.5	194.6	23.9	—	22.7	—	—
1′	—	—	—	—	—	—	—	—	—	—	—	—	—	—	172.8	—	51.9
2′	—	—	—	—	—	—	—	—	—	—	—	—	—	—	43.5	—	—
3′	—	—	—	—	—	—	—	—	—	—	—	—	—	—	25.7	—	—
4′	—	—	—	—	—	—	—	—	—	—	—	—	—	—	22.4	—	—
5′	—	—	—	—	—	—	—	—	—	—	—	—	—	—	22.4	—	—
**NO.**	**95 [7]**	**96 [8]**	**97 [40]**	**98 [42]**	**99 [42]**	**100 [69]**	**101 [69]**	**102 [69]**	**103 [70]**	**104 [70]**	**105 [39]**	**106 [39]**	**107 [71]**	**108 [40]**	**109 [27]**	**110 [40]**	
	**CDCl_3_**	**CDCl_3_**	**CD_3_OD**	**CD_3_OD**	**CD_3_OD**	**C_5_D_5_N**	**C_5_D_5_N**	**C_5_D_5_N**	**CDCl_3_**	**CDCl_3_**	**CDCl_3_**	**CDCl_3_**	**CDCl_3_**	**CD_3_OD**	**C_5_D_5_N**	**CD_3_OD**	
1	79.4	79.4	81.2	136.3	136.2	79.4	76.6	79	41.7	42.5	42.3	42.2	76.4	43.2	127.1	38.7	
2	31.6	31.6	27.6	120.6	120.6	33.3	34.3	29.9	16.7	22.7	16.9	22.8	30.5	22.7	138.3	23.9	
3	34.3	34.3	42.3	29	29.9	33.8	122	42.5	32.7	36.8	32.9	36.9	38.6	26.7	76.2	128.2	
4	148.6	148.8	72.1	82.2	82.2	148.5	136.3	72.3	37.5	148.9	38.7	149.4	206.3	118.1	141.1	134.4	
5	47.7	47.7	54.6	37.9	37.9	53.2	53.6	59.4	149	46.2	152.8	46.7	58.4	53	49	49.9	
6	27	27	70	36.3	36.5	78	79.9	80.9	118.8	27.5	113.7	21.2	76.6	81.1	77.3	82.8	
7	41.5	41.6	51.1	42.5	41.3	45.4	44.8	45.5	39.4	40.5	47.1	47	48.9	54.1	51.7	55.1	
8	29.3	29.3	20.1	25.3	28.1	19.8	17.8	18.1	76.4	76.6	77.2	77.7	20.9	24.1	20.4	23.9	
9	37	37.1	40.7	29.8	30.2	36.4	30.8	34.6	42.6	41.3	42.7	41.4	36.1	41	39.5	40.7	
10	40.4	40.4	41	48.3	37.9	41.2	38.8	40.4	32.6	34.3	33.1	34.7	45.5	39.4	35.7	36.9	
11	148.7	148.6	85.9	14.7	147.9	72.9	72.4	72.1	139.8	142.2	79.2	79.7	138.3	42.2	139.1	41.9	
12	66.4	66.1	24.6	21	170.9	29.6	30.2	30.4	170.3	170.5	175.5	175.5	170.2	182.1	169.4	182.4	
13	111.3	111.2	25.6	10.7	123	31.3	30.7	30.7	121.5	119.9	43.6	42.2	117.2	12.6	115.1	12.6	
14	10.4	10.4	14.3	—	21	15.7	12.6	16.5	22.5	17.6	28.8	18	12.3	18.3	19.6	17.7	
15	107.2	107.2	29.8	—	10.9	107.2	23.1	23.8	28.5	106.6	23.2	106.5	—	139.1	107.5	73.4	
16	—	—	—	—	—	—	—	—	—	—	210.4	210.5	—	—	—	—	
17	—	—	—	—	—	—	—	—	—	—	32	32	—	—	—	—	
1′	171	173	172.5	101.5	101.5	104.4	105.6	105.6	—	—	—	—	—	104.5	104.2	103.8	
2′	21.2	43.7	22.2	75.2	75.2	75.2	74.1	75.2	—	—	—	—	—	74.9	74.4	75.3	
3′	—	25.9	—	78.2	78.2	78.8	78.7	78.8	—	—	—	—	—	78.2	77.5	78.3	
4′	—	22.6	—	71.9	71.9	71.5	71.8	72.4	—	—	—	—	—	71.2	70.8	71.7	
5′	—	22.6	—	77.8	77.8	78.3	78.3	78.5	—	—	—	—	—	78	77.5	77.8	
6′	—	—	—	63	63	62.6	62.8	63	—	—	—	—	—	62.4	61.8	62.8	

Note: The 1″ and 2″ data of compound **97** were 172.7 and 22.5.

**Table 21:** The compound name, molecular formula, and NMR test reagent of polymer sesquiterpene lactones.

No.	Compound Name	Molecular Formula	Solvent	Ref.
**111**	Macrocephadiolide B	C_30_H_34_O_8_	CD_3_OD	[72]
**112**	Ainsliadimer J	C_30_H_34_O_7_	CDCl_3_	[72]
**113**	Ainsliadimer A	C_30_H_34_O_7_	CDCl_3_	[73]
**114**	Macrocephadiolide A	C_30_H_32_O_8_	CDCl_3_	[72]
**115**	Japonicone A	C_32_H_40_O_7_	CDCl_3_	[74]
**116**	Ainsliadimer B	C_30_H_32_O_8_	CDCl_3_	[75]
**117**	Ainsliadimer C	C_30_H_36_O_7_	CDCl_3_	[31]
**118**	Ainsliadimer D	C_30_H_36_O_8_	DMSO	[31]
**119**	Gochnatiolide A	C_30_H_30_O_7_	CDCl_3_	[76,77]
**120**	Ainsliadimer F	C_31_H_36_O_6_	CDCl_3_	[47]
**121**	Ainsliadimer I	C_31_H_34_O_6_	CDCl_3_	[47]
**122**	Ainsliadimer G	C_32_H_36_O_7_	CDCl_3_	[47]
**123**	Ainsliadimer H	C_33_H_38_O_7_	CDCl_3_	[47]
**124**	Gochnatiolide C	C_30_H_30_O_6_	CDCl_3_	[76]
**125**	Gochnatiolide B	C_30_H_30_O_7_	CDCl_3_	[76]
**126**	Gochnatiolide E	C_30_H_30_O_8_	CDCl_3_	[78]
**127**	Gochnatiolide F	C_30_H_34_O_7_	CDCl_3_	[78]
**128**	Macrocephatriolide B	C_45_H_50_O_10_	CDCl_3_	[79]
**129**	Macrocephatriolide A	C_45_H_46_O_10_	CDCl_3_	[79]
**130**	Ainsliatriolides A	C_45_H_48_O_10_	CDCl_3_	[80]
**131**	Ainsliatriolide C	C_45_H_48_O_11_	CDCl_3_	[81]
**132**	Ainsfragolide	C_45_H_46_O_10_	CDCl_3_	[78]
**133**	Ainsliatrimer A	C_45_H_44_O_10_	CDCl_3_	[75]
**134**	Ainsliatrimer B	C_45_H_44_O_10_	CDCl_3_	[75]
**135**	Ainsliatriolides B	C_46_H_50_O_11_	CDCl_3_	[80]

**Table 22:** ^1^H-NMR data of compounds **111**–**118**.

NO.	111 [72]	112 [72]	113 [73]	114 [72]	115 [74]	116 [75]	117 [31]	118 [31]
	CD_3_OD	CDCl_3_	CDCl_3_	CDCl_3_	CDCl_3_	CDCl_3_	CDCl_3_	DMSO
1	3.24, dd, 8.0, 2.3	3.09, ddt, 20.6, 7.7, 4.2	3.15, dd, 12.0, 11.4	3.11, dd, 17.0, 7.0	3.28, dd, 11.7, 3.8	—	—	—
2*α*	2.63, m	2.64–2.54, m	2.38, dd, 13.8, 12.0	2.68, m	1.82, m	—	—	—
2*β*	2.43, m	2.29, tdt, 10.8, 5.0, 3.0	1.88–1.92		1.62, m	—	—	—
3*α*	—	—	—	—	1.65, m	—	—	—
3*β*	—	—	—	—	1.57, m	—	—	—
4	2.40, m	2.29, tdt, 10.8, 5.0, 3.0	—	—	2.45, m	2.68, m	2.50, m	2.37, m
5	2.54, q, 9.4	2.43–2.35, m	2.55, t, 11.4	2.53, t, 10.0	—	3.33, dd, 9.8, 4.3	2.78, dd, 11.4, 4.2	3.37, dd, 11.4, 4.8
6	4.08, t, 9.2	3.95, t, 9.2	4.07, dd, 11.4, 9.0	4.09, dd, 10.0, 8.8	5.37, d, 3.0	4.35, t, 9.8	4.29, t, 10.8	4.39, t, 10.8
7	3.16, m	2.92–2.81, m	2.72, m	2.95, tt, 8.8, 3.0	2.79, dd, 5.5, 3.2	2.80, m	1.82, overlap	2.84, m
8*α*	2.38, m	2.12–1.91, m	2.22–2.24, m	2.34, m	—	2.05, m	2.00, overlap	2.04, m
8*β*	1.49, m	1.55–1.41, m	1.37, dq, 12.6, 6.0	1.49, m	4.89, dd, 5.3, 2.7	2.10, m	1.82, overlap	1.90, m
9*α*	2.63, m	2.64–2.54, m	2.67, m	2.71, m	2.59, dd, 14.8, 3.3	1.88, m	2.00, overlap	1.68, m
9*β*	2.29, td, 12.5, 5.7	1.76, ddd, 14.0, 11.6, 5.0	1.88–1.92	2.16, td, 12.8, 5.0	1.53, dd, 15.1, 2.0	2.05, m	1.72, m	1.81, overlap
11	—	—	—	—	—	—	2.36, m	—
13*α*	6.20, d, 3.2	6.26, dd, 5,1, 3.4	6.19, d, 3.0	6.26, d, 3.2	2.00, m	5.53, d, 3.0	1.27, d, 7.2	6.01, d, 3.0
13*β*	5.68, d, 3.2	5.55, dd, 5.3, 3.1	5.46, d, 3.0	5.57, d, 3.2	1.88, m	6.21, d, 3.0		5.62, d, 3.0
14*α*	5.00, s	5.05, d, 1.1	5.15, s	5.20, s	1.19, s	1.95, m	1.82, overlap	1.81, overlap
14*β*	4.69, s	4.69, s	5.03, s	4.84, s		1.70, m	1.69, m	1.54, m
15*α*	2.09, m	2.12–1.91, m	2.25–2.30	2.16, m	1.11, d, 7.6	3.94, dd, 11.0, 4.0	1.28, d, 7.2	3.88, m
15*β*	1.90, m	1.65–1.56, m	2.16–2.21					3.62, m
1′	3.22, m	2.96, ddq, 12.0, 9.3, 3.2	3.04–3.06	2.97, q, 9.6	—	3.22, m	3.18, t, 9.0	3.27, t, 9.0
2′*α*	2.61, dd, 16.2, 5.5	2.50–2.46, m	2.91, dd, 16.8, 9.6	3.25, dd, 15.6, 10.2	4.60, s	3.23, m	3.22, m	3.27, overlap
2′*β*	2.46, dd, 16.2, 9.4	2.29, tdt, 10.8, 5.0, 3.0	2.45, d, 16.8	2.68, m	—	2.66, m	2.62, d, 18.6	2.49, overlap
3′	—	—	—	—	2.86, d, 1.4	—	—	—
5′	3.26, m	3.09, ddt, 20.6, 7.7, 4.2	3.04–3.06	2.53, t, 10.1	—	3.20, m	3.12, t, 9.6	2.89, t, 9.6
6′*α*	—	—	—	—	3.03, d, 15.5	—	—	—
6′*β*	4.39, t, 9.5	4.27, dd, 11.0, 8.8	4.12, dd, 10.2, 8.4	4.82, dd, 10.1, 9.0	2.08, m	4.12, t, 10.5	4.14, t, 9.6	4.25, t, 9.6
7′	3.07, m	2.92–2.81, m	3.04–3.06	2.89, ddd, 11.9, 9.0, 3.1	2.82, s	3.01, m	2.07, m	3.18, m
8′*α*	2.30, m	2.12–1.91, m	2.25–2.30	1.56, m	—	1.49, m	2.19, overlap	2.27, m
8′*β*	1.53, m	1.55–1.41, m	1.50, dq, 12.6, 3.6	2.28, m	4.21, ddd, 12.4, 8.4, 3.2	2.30, m	1.40, m	1.45, m
9′*α*	2.55, m	2.50–2.46, m	2.16–2.21	2.06, m	2.36, dt, 13.1, 4.1	2.21, m	2.58, m	2.49, br s
9′*β*	2.32, m	2.19, td, 12.5, 5.7	2.57, m	2.68, m	2.00, m	2.62, m	2.12, overlap	2.16, m
10′	—	—	—	—	2.15, m	—	—	—
11′	—	—	—	—	—	—	2.19, overlap	—
13′*α*	6.18, d, 3.2	6.26, dd, 5,1, 3.4	6.31, d, 3.0	6.33, d, 3.2	6.22, d, 3.3	5.57, d, 3.0	1.24, d, 6.6	6.04, d, 3.0
13′*β*	5.67, d, 3.2	5.55, dd, 5.3, 3.1	5.61, d, 3.0	5.57, d, 3.2	5.54, d, 3.1	6.26, d, 3.0		5.68, d, 3.0
14′*α*	5.05, s	5,14, s	4.96, s	5.19, s	1.05, d, 7.3	5.09, s	5.06, s	5.83, s
14′*β*	4.99, s	4.99, s	4.59, s	5.10, s		4.73, s	4.70, s	5.03, s
15′*α*	2.97, ddd, 18.3, 9.0, 5.3	2.72, ddd, 13.2, 4.8, 3.0	2.05, dd, 14.4, 5.4	2.80, m	1.67, d, 1.5	2.15, m	2.12, overlap	2.20, m
15′*β*	2.74, ddd,18.3, 9.0, 6.2	2.64–2.54, m	2.01, dd, 14.4, 7.8	2.25, m		2.06, m	2.05, m	1.85, m

Note: The ^1^H-NMR data of 2″ of **115** were 2.08, s.

**Table 23:** ^1^H-NMR data of compounds **119**–**127**.

NO.	119 [76]	120 [47]	121 [47]	122 [47]	123 [47]	124 [76]	125 [76]	126 [78]	127 [78]
	CDCl_3_	CDCl_3_	CDCl_3_	CDCl_3_	CDCl_3_	CDCl_3_	CDCl_3_	CDCl_3_	CDCl_3_
4	—	2.52, dd, 7.2, 4.2	2.56, dd, 7.1, 4.2	2.63, m	2.62, m	—	—	—	2.46, m
5	3.92, d, 10.4	2.80, dd, 11.1, 4.1	2.91, dd, 11.2, 4.2	3.52, dd, 12.3, 5.3	3.52, dd, 11.3, 4.1	3.55, d, 10.4	3.89, d, 10.8	3.62, d, 10.0	3.03, dd, 1.5, 10.2
6	3.76, t, 9.9	4.32, dd, 11.0, 9.6	4.33, dd, 11.1, 9.7	4.38, dd, 11.2, 9.8	4.36, dd, 11.3, 9.7	3.92, d, 9.9	4.36, t, 10.7	4.59, d, 10.0	3.75
7	3.88, m	1.84, m	2.75, mH	2.87, m	2.86, m	3.23–3.29, m	2.86, m	—	3.75
8*α*	2.34–2.44, m	2.05, m	2.10, m	2.16, m	2.11, m	2.32–2.44, m	2.04–2.11, m	3.41	2.39, m
8*β*	1.60–1.67, m	1.85, m	2.02, m	2.08, m	2.03, m	1.68–1.75, m	2.04–2.11, m	2.66	2
9*α*	2.06–2.16, m	1.97, m	1.71, m	2.00, m	1.95, m	2.00–2.07, m	2.04–2.11, m	2.22	2.07, m
9*β*	2.06–2.16, m	1.70, m	1.68, m	1.73, m	1.73, m	1.61–1.68, m	1.84–2.01, m	2.09	2.03
10	—	—	—	—	—	2.74, m	—	—	—
11	—	2.35, m	—	—	—	—	—	—	—
13*α*	6.21, d, 3.7	1.27, d, 6.9	6.20, d, 3.2	6.23, d, 3.3	6.21, d, 3.3	6.28, d, 3.4	6.23, d, 3.4	4.43, dd, 13.3, 19.5	6.18, d, 3.2
13*β*	5.49, d, 3.3		5.52, d, 3.1	5.56, d, 3.0	5.54, d, 3.1	5.57, d, 3.1	5.56, d, 3.1		5.45, d, 3.2
14*α*	1.87–2.00, m	1.86, m	1.86, m	2.12, m	2.09, m	2.00–2.07, m	1.84–2.01, m	1.95	1.87
14*β*	1.87–2.00, m	1.69, m	1.69, m	1.94, m	1.93, m	1.84–1.97, m	1.74, m		
15*α*	6.22, br s	1.27, d, 7.1	1.30, d, 7.2	3.94, dd, 9.5, 2.2	3.96, dd, 9.6, 2.3	6.18, br s	6.24, br s	6.26, s	1.23, d, 7.6
15*β*	6.03, br s			3.74, dt, 9.5, 3.2	3.76, dd, 9.7, 3.1	6.00, br s	6.16, br s	6.05, s	
1′	3.25, t, 9.6	3.22, m	3.22, t, 9.0	3.22, m	3.24, t, 9.3	3.23–3.29, m	3.27, m	3.27	3.17, t, 8.9
2′*α*	3.37, t, 10.0	3.22, m	3.23, m	3.22, m	2.68, m	3.33–3.40, m	3.38, m	3.32	3.23, m
2′*β*	2.63, m	2.63, m	2.64, m	2.63, m	2.62, m	2.62–2.66, m	2.70, m	2.68	2.61
5′	3.34, t, 10.5	3.20, d, 4.9	3.19, t, 8.7	3.25, t, 10.2	3.17, t, 9.3	3.33–3.40, m	3.28, m	3.39	3.11, t, 9.4
6′	4.24, t, 9.2	4.16, t, 9.4	4.17, t, 9.2	4.19, t, 9.2	4.18, t, 9.8	4.21, t, 9.5	4.21, m	4.26, t, 9.8	4.20, t, 9.4
7′	3.08, m	3.00, m	3.01, m	3.05, m	3.03, m	3.06, m	3.03, m	3.08, m	2.15
8′*α*	2.34–2.44, m	2.31, m	2.35, m	2.35, m	2.33, m	2.32–2.44, m	2.34, m	2.35, m	2.20, m
8′*β*	1.49–1.57, m	1.47, m	1.48, m	1.51, m	1.51, m	1.51, m	1.50, m	1.54, m	1.42, m
9′*α*	2.66, br s	2.64, m	2.65, m	2.65, m	2.64, m	2.62–2.66, m	2.66, s	2.66	2.57
9′*β*	2.34–2.44, m	2.20, m	2.22, m	2.24, m	2.23, m	2.26, m	2.25, m	2.26	2.13
11′	—	—	—	—	—	—	—	—	2.25, m
13′*α*	6.30, d, 3.4	6.26, d, 3.4	6.27, d, 3.4	6.29, d, 3.4	6.27, d, 3.4	6.29, d, 3.4	6.28, d, 3.4	6.29, d, 3.0	1.26, d, 7.0
13′*β*	5.64, d, 3.1	5.58, d, 3.0	5.58, d, 3.0	5.60, d, 3.0	5.59, d, 3.0	5.60, d, 3.0	5.60, d, 3.1	5.65, d, 3.0	
14′*α*	5.10, s	5.09, br s	5.09, br s	5.12, br s	5.11, br s	5.08, s	5.12, s	5.11, s	5.05, s
14′*β*	4.71, s	4.72, br s	4.72, br s	4.75, br s	4.74, br s	4.73, s	4.75, s	4.75, s	4.67, s
15′*α*	2.06–2.16, m	2.13, m	2.11, m	2.12, m	2.12, m	2.00–2.07, m	2.04–2.11, m	2.15	2.1
15′*β*	1.87–2.00, m	2.02, m	1.97, m	2.07, m	2.07, m	1.84–1.97, m	2.04–2.11, m	1.92	1.85
1″	—	—	—	3.29, s	3.42, dd, 7.0, 3.5	—	—	—	—
2″	—	—	—	—	1.06, t, 7.0	—	—	—	—

**Table 24:** ^1^H-NMR data of compounds **128**–**135**.

NO.	128 [79]	129 [79]	130 [80]	131 [81]	132 [78]	133 [75]	134 [75]	135 [80]	NO.	128 [79]	129 [79]	130 [80]	131 [81]
	CDCl_3_	CDCl_3_	CDCl_3_	CDCl_3_	CDCl_3_	CDCl_3_	CDCl_3_	CDCl_3_		CDCl_3_	CDCl_3_	CDCl_3_	CDCl_3_
1	3.06, m	—	—	—	—	—	—	—	1′	2.72, m	3.07, dd, 10.6, 5.9	3.28, m	3.28, m
2	2.51–2.58, m	—	—	—	—	—	—	—	2′*α*	3.43, q, 7.3	3.36, dt, 8.5, 5.9	3.27, m	3.26, m
4	2.22, m	—	2.66, m	—	—	—	—	—	2′*β*			2.62, m	2.63, m
5	2.45, m	3.91, d, 10.3	3.23, dd, 11.0, 3.7	3.40, m	3.94, d, 9.0	3.43, d, 10.3	3.87, d, 10.3	3.07, m	5′	2.47, t, 11.1	3.43, m	3.34, m	3.26, m
6	3.92, t, 9.2	3.76, dd, 10.3, 10.3	4.21, dd, 11.0, 9.8	4.47, t, 10.0	3.80, d, 9.0	3.78, t, 10.3	3.64, t, 10.3	4.48, t, 10.5	6′	4.32, dd, 11.1, 8.7	4.25, dd, 11.0, 8.6	4.12, t, 9.4	4.12, t, 10.0
7	2.97, m	3.85, m	3.00, m	3.02, m	3.89, m	3.21, m	3.78, m	2.66, m	7′	2.79, m	2.99, m	3.12, m	3.12, m
8*α*	2.20, m	2.36, m	2.06, m	2.07, m	2.4	1.82, m	1.80, m	1.91, m	8′*α*	2.24, m	2.30, m	2.32, m	2.28, m
8*β*	1.44, m	1.56, m	2.02, m	1.98, m	1.61	2.35, m	2.34, m	1.95, m	8′*β*	1.50, m	1.46, m	1.48, m	1.48, m
9*α*	2.56, m	2.05, m	2.05, m	2.03, m	2.08, m	2.00, m	2.05, m	1.67, m	9′*α*	2.64, m	2.69, m	2.61, m	2.61, m
9*β*	2.18, m	1.92, m	2.01, m	1.75, m	1.95, m	2.38, m	2.49, m	1.92, m	9′*β*	1.97, td, 12.8, 4.5	2.18, m	2.27, m	2.21, m
10	—	—	—	—	—	2.77, m	—	—	13′*α*	6.23, d, 3.3	6.22, d, 3.2	6.15, d, 3.2	6.17, d, 3.4
13*α*	6.15, d, 3.2	6.16, d, 3.4	6.15, d, 3.2	6.11, d, 3.2	6.21, d, 3.2	5.57, d, 3.0	5.49, d, 3.4	5.43, d, 3.3	13′*β*	5.52, d, 3.3	5.58, d, 3.2	5.50, d, 3.2	5.52, d, 3.4
13*β*	5.49, d, 3.2	5.45, d, 3.4	5.50, d, 3.2	5.47, d, 3.2	5.48, d, 3.2	6.25, d, 3.0	6.19, d, 3.4	6.10, d, 3.3	14′*α*	5.37, br s	5.18, br s	5.08, br s	5.08, s
14*α*	4.96, br s	1.98, m	1.84, m	1.84, m	2	2.55, m	2.65, m	1.78, td, 13.9, 3.7	14′*β*	5.13, br s	5.10, br s	4.71, br s	4.74, s
14*β*	4.66, br s	1.88, m	1.70, m	1.73, m	1.93, m	1.64, m	1.93, m	1.56, m	15′*α*	2.04, m	2.28, m	2.18, m	2.13, m
15*α*	1.98, m	6.17, br s	1.91, m	2.15, m	6.22, br s	5.97, s	5.99, s	2.51, m	15′*β*	1.85, td, 12.8, 4.7	1.65, td, 14.3, 3.7	2.05, m	2.01, m
15*β*	1.43, m	5.97, br s	1.84, m	1.86, m	6.02, br s	6.26, s	6.30, s	2.13, m					
**NO.**	**132 [78]**	**133 [75]**	**134 [75]**	**135 [80]**	**NO.**	**128 [79]**	**129 [79]**	**130 [80]**	**131 [81]**	**132 [78]**	**133 [75]**	**134 [75]**	**135 [80]**
	**CDCl_3_**	**CDCl_3_**	**CDCl_3_**	**CDCl_3_**		**CDCl_3_**	**CDCl_3_**	**CDCl_3_**	**CDCl_3_**	**CDCl_3_**	**CDCl_3_**	**CDCl_3_**	**CDCl_3_**
1′	3.10, m	—	—	3.06, m	1″	3.10, m	3.11, td, 8.7, 4.5	3.06, m	3.09, m	3.14, m	3.14, m	3.13, m	—
2′*α*	—	—	—	3.07, m	2″*α*	2.45–2.55, m	2.56, m	2.48, m	2.47, m	2.6	3.11, m	3.07, m	—
2′*β*	3.39, m	—	—	2.71, m	2″*β*		2.43, m	2.46, m	2.29, m	2.48	2.58, m	2.60, m	—
5′	3.46, t, 10.7	3.19, d, 10.0	3.17, d, 9.6	3.24, t, 9.3	4″	2.62, m	2.52, m	2.29, m	2.29, m	2.55	—	—	—
6′	4.29, dd, 10.7, 8.7	4.36, t, 10.0	4.35, t, 9.6	4,23, t, 9.3	5″	2.55, m	2.40, m	2.51, m	2.52, m	2.44	2.95, m	2.94, t, 9.6	2.37, m
7′	3.04	2.65, m	2.65, m	2.89, m	6″	3.96, t, 9.1	3.98, dd, 9.3, 9.3	3.94, t, 9.1	3.89, t, 10.0	4.02, t, 9.2	4.22, t, 9.5	4.22, t, 9.6	4.19, dd, 11.3, 8.8
8′*α*	2.32	1.99, m	1.99, m	1.50, m	7″	3.04	2.96, m	2.96, m	3.03, m	2.99	2.99, m	3.00, m	2.77, m
8′*β*	1.5	2.15, m	2.08, m	2.31, m	8″*α*	2.19, m	2.29, m	2.27, m	2.16, m	2.33	1.50, m	1.52, m	1.56, m
9′*α*	2.73, m	1.80, m	1.78, m	2.16, m	8″*β*	1.47, m	1.46, m	1.41, m	1.39, m	1.5	2.34, m	2.31, m	2.18, m
9′*β*	2.22	1.94, m	2.10, m	2.66, m	9″*α*	2.56, m	2.55, m	2.56, m	2.57, m	2.59	2.20, m	2.31, m	2.27, m
13′*α*	6.27, d, 3.2	5.48, d, 3.0	5.48, d, 3.2	5.59, d, 3.2	9″*β*	2.18, m	2.20, m	2.18, m	2.19, m	2.22	2.61, m	2.63, m	2.35, m
13′*β*	5.62, d, 3.2	6.10, d, 3.0	6.11, d, 3.2	6.28, d, 3.2	13″*α*	6.12, d, 3.2	6.23, d, 3.2	6.19, d, 3.2	6.17, d, 3.2	6.28, d, 3.0	6.27, d, 3.0	6.28, d, 3.2	6.16, d, 3.4
14′*α*	5.22, s	2.10, m	2.09, m	5.13, br s	13″*β*	5.47, d, 3.2	5.54, d, 3.2	5.50, d, 3.2	5.52, d, 3.2	5.57, d, 3.0	5.59, d, 3.0	5.59, d, 3.2	5.45, d, 3.4
14′*β*	5.14, s	1.80, m	1.79, m	4.79, br s	14″*α*	4.99, br s	4.98, br s	4.96, br s	4.94, s	5.02, s	5.09, s	5.10, s	4.93, br s
15′*α*	2.32	2.17, m	2.17, m	1.90, m	14″*β*	4.65, br s	4.73, br s	4.63, br s	4.59, s	4.77, s	4.76, s	4.77, s	4.89, br s
15′*β*	1.67	1.85, m	1.99, m	2.08, m	15″*α*	2.29, m	2.29, m	1.76, m	1.68, m	2.32	2.30, m	2.30, m	2.18, m
					15″*β*	1.67, ddd, 13.9, 7.5, 5.8	1.96, m	1.59, m	1.48, m	2.03	2.06, m	2.30, m	1.99, m

**Table 25:** ^13^C-NMR data of compounds **111**–**127**.

NO.	111 [72]	112 [72]	113 [73]	114 [72]	115 [74]	116 [75]	117 [31]	118 [31]	119 [77]	120 [47]	121 [47]	122 [47]	123 [47]	124 [76]	125 [76]	126 [78]	127 [78]
	CD_3_OD	CDCl_3_	CDCl_3_	CDCl_3_	CDCl_3_	CDCl_3_	CDCl_3_	DMSO	CDCl_3_	CDCl_3_	CDCl_3_	CDCl_3_	CDCl_3_	CDCl_3_	CDCl_3_	CDCl_3_	CDCl_3_
1	41	38.3	41.8	36.6	80.7	173.6	171.8	172.7	170.6	172	171.6	173.2	173.1	171	169	169.6	174
2	45.3	45.9	38.1	39.8	26	140.7	140.3	140.5	143.2	140.2	140.4	140.6	140.8	142.1	142.7	142.9	140.6
3	221	218.5	90.5	213.9	29.7	207.3	208.5	206.1	194.1	208.5	208.4	206.3	206.5	193.8	193.6	193.8	210
4	51.6	52.1	89.1	90.8	38.2	53.6	46.9	53.6	142	47	47	52	51.8	141.8	141.1	142.1	45
5	49.1	48.8	53.1	49.1	149.5	49	54.9	46.9	47.3	54.8	55.1	48.3	48.5	51	49.7	46.5	52.6
6	90.1	87.9	82.9	81.7	118.1	82.3	82.6	82.2	83.6	82.6	82.6	82.6	82.6	82.7	81.2	84	85
7	45.1	44.4	49	44.4	42.2	51.6	54.7	50.8	43.4	54.9	51.6	51.6	51.7	43.7	52.4	166	43.3
8	32.7	31.7	31.3	31.9	75.3	21	22.5	20.7	35.3	22.5	21	21	21	25.3	20.8	21.7	23
9	39	37.9	37.8	39.2	39.8	36	36.3	36.7	38.4	36.2	36.1	36.2	36.2	27.7	35.9	35.2	35
10	150.9	148.6	147.5	148	38.5	68.3	68.2	67.3	71.1	68.1	68.3	68.4	68.4	33.3	68.2	69.3	71.2
11	141.1	139	139.5	138.4	56.8	139.3	41.9	140	140.3	41.9	139.4	139.5	139.6	139.3	138.8	126	140.6
12	171.9	169.7	170	169.1	178.8	169.7	178.5	169.6	169.9	178.6	170	170.1	170.1	168.4	169.8	172.8	170
13	121.6	121.4	119.7	121.8	36.4	119.3	12.6	118.4	119.6	12.6	119.1	119.1	119.1	120.7	119.2	54.6	119.5
14	113.4	113.8	113.8	114.4	21.6	36.2	36.3	36	28.3	36.3	36.3	36	36	31.2	36.3	37.3	38.6
15	23.6	22.8	33.1	28.6	23	60.6	14.3	57.5	122.5	14.3	14.2	69.2	67.2	120.9	122.4	122.3	16.5
1′	41.1	39.6	50.1	36.1	62.3	40	40	38.7	39.9	40	40	40	40	40	40.1	39.8	39.7
2′	36.5	44.4	46.9	34.9	81.9	44.7	44.8	39.3	44.9	44.7	44.7	44.8	44.8	45.1	44.7	44.7	44.7
3′	175.1	215.3	224.9	172.4	56.2	222	222.1	220.4	219.7	222.3	222.1	222.5	222.4	220.4	222.1	219.4	219.8
4′	212.5	80.1	62.2	113.2	134.2	51	50.9	50.1	51	50.9	50.9	50.9	50.9	51	51.1	51.1	50.7
5′	57.4	51.4	38.9	47.3	136.4	49.6	50	50.8	49.1	49.6	49.5	49.6	49.7	49.4	49.6	48.8	49.7
6′	86	82.2	84	81.6	26	83.8	83.6	83.6	84.4	83.9	83.9	83.9	83.8	84.1	83.9	84.3	84
7′	44.4	40.1	43.7	45.3	45.3	43.5	47.8	42.5	43.5	43.5	43.5	43.4	43.5	43.5	43.5	43.6	47.8
8′	31.1	31.5	32.8	31.9	82.5	32	33	31.1	31.9	31.9	31.9	32	32	32	32	31.9	32.9
9′	37.9	36.5	38.9	40.1	36.1	39.5	39.8	39.2	39.4	39.5	39.5	39.5	39.5	39.5	39.4	39.5	39.8
10′	148.2	147.2	150	145.8	29.8	150.1	150.6	151.2	150.1	150.1	150.1	150.2	150.2	150.6	150.1	150.1	150.4
11′	140.8	138.7	138.6	138.8	139.4	138.5	41.8	139.3	138.1	138.5	138.4	138.6	138.5	138.6	138.4	138.1	41.8
12′	171.3	169.6	169	169.8	170	169.3	177.5	169.2	170.4	169.3	169.3	169.4	169.2	169.8	169.5	170.7	179
13′	121.4	121.4	122	120.8	119.5	121.7	13.4	120.6	121.8	121.6	121.7	121.5	121.5	121.6	121.8	122.7	13.3
14′	116	114.3	113.7	116.2	17	114.2	113.6	113.1	114.1	114.1	114.1	114.1	114.1	114	114.2	114.2	113.6
15′	42.9	31.2	26.3	37.1	14.3	25.8	26	25.7	23	25.8	25.8	25.9	25.9	26.2	25.8	27.6	28.4
1″	—	—	—	—	170	—	—	—	—	—	—	59.2	66.8	—	—	—	—
2″	—	—	—	—	21.2	—	—	—	—	—	—	—	15	—	—	—	—

**Table 26:** ^13^C-NMR data of compounds **128**–**135**.

NO.	128 [79]	129 [79]	130 [80]	131 [81]	132 [78]	133 [75]	134 [75]	135 [80]	NO.	128 [79]	129 [79]	130 [80]	131 [81]	132 [78]	133 [75]	134 [75]	135 [80]
	CDCl_3_	CDCl_3_	CDCl_3_	CDCl_3_	CDCl_3_	CDCl_3_	CDCl_3_	CDCl_3_		CDCl_3_	CDCl_3_	CDCl_3_	CDCl_3_	CDCl_3_	CDCl_3_	CDCl_3_	CDCl_3_
1	40.1	170.4	173.4	172.9	170.1	173.8	172.4	149.6	1′	44.9	46.5	40.2	39.9	46.4	171.9	172.3	40
2	44.4	142.8	141.6	139.4	142.7	138.2	139.1	134.8	2′	42.7	48.6	44.6	44.6	48.6	140.3	140	44
3	218.7	193.6	207.8	205.2	193.6	193.9	194.2	115.4	3′	215	218.3	222.2	223	218.2	207.6	207.3	222
4	51.2	142.3	49.2	79	142.1	141.4	141.6	79.4	4′	80.9	52.8	51.2	51	52.7	52.3	51.9	52.7
5	49.1	47.6	51.2	53.6	47.5	51.2	46.9	56.9	5′	50.4	45.9	49.5	50.2	45.8	58.1	58.3	48.2
6	88.1	84	82.9	80.3	83.9	83.2	84	80.3	6′	81.9	83.7	84.2	84.2	83.6	80.7	80.6	83.9
7	44.2	43.7	51.5	51.4	43.6	44	43.5	50.5	7′	46.7	45.2	43.3	43.4	45	52.2	52.2	44.2
8	31.8	23.1	21	21.9	23	24.9	22.9	21.5	8′	31.3	32.1	31.9	31.8	32	21.2	21.1	32
9	38.1	35.2	35.8	36.3	35.1	28.9	34.2	36.9	9′	39.4	39.8	39.6	39.7	39.7	36.6	36.6	39.9
10	148.8	71.2	68.7	68	71.2	34.6	72.6	67.9	10′	146.2	148.9	150.4	150.1	148.8	68.3	68.3	149.6
11	139.4	140.6	140.1	139.3	140.4	139.3	140.2	139.9	11′	138.7	138.4	139.1	138.8	138.2	138.8	138.6	138.5
12	169.8	170.1	170.4	170.3	170	169.4	169.8	170.7	12′	169.8	170.9	169.5	169.6	170.8	169.4	169.3	169.9
13	120.8	119.6	118.7	118.5	119.5	120.5	119.6	118.4	13′	121.1	122.4	121	121.3	122.3	119.3	119.5	122.1
14	113.6	38.6	36.5	36.7	38.5	27.1	38.2	37.1	14′	114.8	114.6	114.2	114.5	114.5	36.7	36.6	114.9
15	23.6	121.6	25.2	31.7	121.7	122	123	27.8	15′	32.2	27	26.2	25.9	26.9	28.6	31.2	26.1
**NO.**	**128 [79]**	**129 [79]**	**130 [80]**	**131 [81]**	**132 [78]**	**133 [75]**	**134 [75]**	**135 [80]**									
	**CDCl_3_**	**CDCl_3_**	**CDCl_3_**	**CDCl_3_**	**CDCl_3_**	**CDCl_3_**	**CDCl_3_**	**CDCl_3_**									
1″	40.0	40.4	39.8	39.6	40.3	39.9	39.9	46.5									
2″	44.1	43.9	44.9	44.9	43.8	44.6	44.5	45.2									
3″	218.4	218.2	218.3	218.5	218.3	218.8	218.8	109.4									
4″	49.3	49.0	51.6	51.1	48.9	50.5	50.5	95.2									
5″	48.6	49.9	46.6	46.2	49.8	50.3	50.4	54.5									
6″	88.2	87.0	89.0	89.0	86.9	84.1	84.0	79.4									
7″	44.0	44.8	44.1	43.9	44.7	43.7	43.7	43.7									
8″	31.4	31.3	31.6	31.1	31.2	31.9	31.9	27.6									
9″	38.1	36.8	38.7	38.8	36.7	39.6	39.6	27.1									
10″	148.7	148.2	148.7	148.8	148.1	150.0	149.9	146.1									
11″	139.3	139.2	139.1	138.7	139.1	138.6	138.5	139.1									
12″	169.8	169.6	169.9	169.8	169.5	169.4	169.4	169.5									
13″	120.6	121.2	120.9	121.1	121.2	121.6	121.7	120.5									
14″	113.6	114.3	113.3	113.1	114.2	114.4	114.5	116.3									
15″	29.6	32.8	22.5	20.9	32.8	25.8	25.8	22.7									

**Table 27:** The compound name, molecular formula, and test reagent of other sesquiterpenoids.

No.	Compound Name	Molecular Formula	Solvent	Ref.
**136**	1-Oxo-bisabola-2-ene-12-ol	C_15_H_26_O_2_	CDCl_3_	[82]
**137**	Pubescone	C_14_H_22_O_2_	CDCl_3_	[83]
**138**	Ainsliaea acid A	C_16_H_22_O_3_	CD_3_OD	[67]
**139**	Curzerenone	C_15_H_18_O_2_	CDCl_3_	[84]
**140**	1-O-Acetyl-6-O-isobutyrylbritannilactone	C_20_H_30_O_6_	CDCl_3_	[85]
**141**	6*α*-(3-Methylvaleryloxy)-1-hydroxy-4*α*H-1,10-secoeudesma-5(10),11(13)-dien-12,8*β*-olide	C_21_H_32_O_5_	CDCl_3_	[86]
**142**	Kobusone	C_14_H_21_O_2_	CDCl_3_	[87]
**143**	10-Hydroxy-6,10-epoxy-7(14)-isodaucane	C_15_H_24_O_2_	CDCl_3_	[88]
**144**	Clovane-2*β*,9*α*-diol	C_15_H_26_O_2_	CDCl_3_	[89]
**145**	Caryolane-1,9*β*-diol	C_15_H_26_O_2_	CDCl_3_	[89]

**Table 28:** ^1^H-NMR data of compounds **136**–**145**.

NO.	136 [82]	137 [83]	138 [67]	139 [84]	140 [85]	141 [86]	142 [87]	143 [88]	144 [89]	145 [89]
	CDCl_3_	CDCl_3_	CD_3_OD	CDCl_3_	CDCl_3_	CDCl_3_	CDCl_3_	CDCl_3_	CDCl_3_	CDCl_3_
1*α*	—	—	5.90, dd, 17.8, 11.2	5.78, dd, 17.6, 10.6	3.85–4.00, m	3.52, m	—	—	—	—
1*β*	—	1.66–1.63, m	—	—		3.44, m	1.93, ddd, 10.4, 8.8, 1.2	—	—	—
2*α*	5.86, m	1.76–1.63, m	5.34, dd, 17.8, 1.1	4.74–4.99, m	1.34–1.45, m	1.32, m	1.67, m	1.82	3.79, dd, 10.5, 5.5	2.22, ddd, 12, 10, 8.5
2*β*			5.25, dd, 18.0, 1.2		1.21–1.31, m	1.13, m	1.54, m	1.37		
3*α*	—	2.53–2.48, m	4.90, m	4.74–4.99, m	1.21–1.31, m	1.32, m	2.15, dt, 13.2, 3.6	1.87	1.51, dd, 11.5, 10	1.49, dd, 10, 9
3*β*	—				0.97–1.08, m	1.00, m	0.94, td, 13.2, 4.4	1.27	1.71, dd, 11.5, 5.5	1.54, t, 10
4	2.32, m	—	—	—	2.60–2.76, m	2.70, m	—	1.58	—	—
5*α*	1.92, m	1.05–1.03, m	2.79, br s	3.00, s	—	—	—	1.56	—	1.89, ddd, 12, 9, 6
5*β*	1.78, m				—	—	2.69, dd, 10.0, 5.2	—	1.42, m	—
6*α*	2.15, m	0.72–0.67, m	—	—	5.19, d, 1.7	5.23, d, 1.7	2.40, m	3.98	1.32, m	1.39, m
6*β*	—	—	4.64, br s	—	—	—	1.44, m	—	1.35, m	1.53, m
7*α*	2.33, m	—	2.02, m	—	3.41–3.49, m	3.50, m	2.55, dd, 6.4, 2.0	—	1.11, m	1.15, m
7*β*	—	—	—	—	—	—	2.53, d, 6.4	—	1.50, m	1.42, m
8*α*	1.26, m	2.05, dd, 8.8, 3.2	1.96, m	—	4.90–4.98, m	5.01, m	—	2.49	—	—
8*β*			1.43, m	—	—	—	—	2.35	—	—
9*α*	1.34, m	2.14–2.11, m	1.86, m	2.83, AB system, 17.6	2.60–2.76, m	2.75, dd, 16.2, 2.1	3.05, td, 8.8, 8.8	2.02	—	3.44, t, 3
9*β*		2.02–1.99, m			2.45–2.56, m	2.51, dd, 16.2, 2.3	—	1.76	3.32, br s	—
10*α*	1.39, m	1.88–1.84, m	—	—	—	—	2.06, dd, 10.4, 8.8	—	1.64, m	1.77, ddt, 15, 5, 3
10*β*	1.12, m	—	—	—	—	—	1.66, d, 10.4	—	1.99, m	2.04, dddd, 15, 12.5, 5.5, 3
11*α*	1.62, m	1.80–1.77, m	—	—	—	—	—	1.51	1.07, m	1.51, m
11*β*	—	—	2.36, m	—	—	—	—	—	1.66, m	1.64, td, 12.5, 5
12*α*	3.50, dd, 10.6, 5.8	0.92, d, 6.8	—	7.08, br s	—	—	1.30, s	0.89, d, 6.6	0.91, br d, 12.5	1.42, d
12*β*	3.42, dd, 10.6, 5.8		—		—	—			1.56, d, 12.5	1.47, d
13*α*	0.91, d, 6.7	0.92, d, 6.8	1.24, d, 7.0	2.16, br s	6.37, d, 2.9	6.36, d, 2.6	1.02, s	0.92, d, 6.6	0.86, s	1.00, s
13*β*					5.94, d, 2.3	6.02, d, 2.3				
14	0.79, d, 6.8	2.18, s	—	1.17, s	1.80, s	1.82, s	1.02, s	1.15, s	1.04, s	1.02, s
15*α*	1.93, br s	—	1.67, s	1.83, br s	0.86, d, 6.9	0.90, d, 7.0	—	4.76, br s	0.96, s	0.93, s
15*β*		—					—	4.70, br s		
2′*α*	—	—	—	—	2.45–2.56, m	2.32, m	—	—	—	—
2′*β*	—	—	—	—		2.11, m	—	—	—	—
3′	—	—	—	—	1.15, d, 6.9	1.87, m	—	—	—	—
4′*α*	—	—	—	—	1.15, d, 6.9	1.37, m	—	—	—	—
4′*β*	—	—	—	—	—	1.26, m	—	—	—	—
5′	—	—	—	—	—	0.92, t, 7.5	—	—	—	—
6′	—	—	—	—	—	0.94, d, 7.0	—	—	—	—

Note: The ^1^H-NMR data of 2″ of compound **140** were 2.04, s.

**Table 29:** ^13^C-NMR data of compounds **136**–**145**.

NO.	136 [82]	137 [83]	138 [67]	139 [84]	140 [85]	141 [86]	142 [87]	143 [88]	144 [89]	145 [89]	NO.	140 [85]
	CDCl_3_	CDCl_3_	CD_3_OD	CDCl_3_	CDCl_3_	CDCl_3_	CDCl_3_	CDCl_3_	CDCl_3_	CDCl_3_		CDCl_3_
1	201.4	34.4	135.8	145.5	64.2	61.7	51.1	54.5	44.6	70.7	1′	176.9
2	127.2	24.8	117.9	115.7	26.5	30.7	26.2	26.4	80.8	38	2′	34.6
3	161.4	41.7	115.5	113	31	30.3	38.8	30.6	47.5	34	3′	18.7
4	30.6	208.7	144.4	141.1	33	32.9	58.6	57.6	37.1	35	4′	18.8
5	22.6	29.9	61.5	64.1	132.1	131.9	61.3	58.9	50.6	43.8	1″	171.2
6	50.1	46.4	82.2	194	69	68.8	24.5	86	20.7	20.3	2″	21
7	30.9	214.2	43.3	120.2	42.9	42.7	37.4	144.8	33.2	35.3		
8	34.9	32.8	25	165.6	74.9	75.6	213.8	33.2	34.7	39.3	**NO.**	**141 [86]**
9	24.9	22.9	34.7	33.6	34.1	34.1	52.3	35.9	75.1	72.1		**CDCl_3_**
10	33.3	27.9	52.3	42.9	133.6	133.3	35	105.1	26	28.1	1′	173.4
11	35.8	30.9	43.8	119.3	136.3	136	34.2	34.1	26.4	33.3	2′	41.3
12	68.4	19.5	179.2	139.6	169.5	170.5	16	21.5	35.6	42.4	3′	31.6
13	16.7	19.2	15.4	9.1	124.9	125.1	29.1	20.4	25.4	20.8	4′	28.8
14	15.8	29.9	180	24.9	20.5	19.9	22	21.6	31.4	30.5	5′	10.6
15	24.2	—	21.9	25	18.6	18.1	—	108.4	28.4	26.7	6′	18.7

## Data Availability

Not applicable.
